# Impairment of TrkB-PSD-95 Signaling in Angelman Syndrome

**DOI:** 10.1371/journal.pbio.1001478

**Published:** 2013-02-12

**Authors:** Cong Cao, Mengia S. Rioult-Pedotti, Paolo Migani, Crystal J. Yu, Rakesh Tiwari, Keykavous Parang, Mark R. Spaller, Dennis J. Goebel, John Marshall

**Affiliations:** 1 Department of Molecular Pharmacology, Physiology, and Biotechnology, Brown University, Providence, Rhode Island, United States of America; 2 Institute of Neuroscience, The Second Affiliated Hospital of Soochow University, Soochow University, Suzhou, China; 3 Dipartimento di Scienze della Vita e dell'Ambiente, Università Politecnica delle Marche, Ancona, Italy; 4 Department of Biomedical and Pharmaceutical Sciences, College of Pharmacy, University of Rhode Island, Kingston, Rhode Island, United States of America; 5 Norris Cotton Cancer Center and Department of Pharmacology and Toxicology, Dartmouth Medical School, Lebanon, New Hampshire, United States of America; 6 Department of Anatomy and Cell Biology, Wayne State University, Detroit, Michigan, United States of America; University of Pennsylvania, United States of America

## Abstract

Angelman syndrome (AS) is a neurodevelopment disorder characterized by severe cognitive impairment and a high rate of autism. AS is caused by disrupted neuronal expression of the maternally inherited Ube3A ubiquitin protein ligase, required for the proteasomal degradation of proteins implicated in synaptic plasticity, such as the activity-regulated cytoskeletal-associated protein (Arc/Arg3.1). Mice deficient in maternal Ube3A express elevated levels of Arc in response to synaptic activity, which coincides with severely impaired long-term potentiation (LTP) in the hippocampus and deficits in learning behaviors. In this study, we sought to test whether elevated levels of Arc interfere with brain-derived neurotrophic factor (BDNF) TrkB receptor signaling, which is known to be essential for both the induction and maintenance of LTP. We report that TrkB signaling in the AS mouse is defective, and show that reduction of Arc expression to control levels rescues the signaling deficits. Moreover, the association of the postsynaptic density protein PSD-95 with TrkB is critical for intact BDNF signaling, and elevated levels of Arc were found to impede PSD-95/TrkB association. In Ube3A deficient mice, the BDNF-induced recruitment of PSD-95, as well as PLCγ and Grb2-associated binder 1 (Gab1) with TrkB receptors was attenuated, resulting in reduced activation of PLCγ-α-calcium/calmodulin-dependent protein kinase II (CaMKII) and PI3K-Akt, but leaving the extracellular signal-regulated kinase (Erk) pathway intact. A bridged cyclic peptide (CN2097), shown by nuclear magnetic resonance (NMR) studies to uniquely bind the PDZ1 domain of PSD-95 with high affinity, decreased the interaction of Arc with PSD-95 to restore BDNF-induced TrkB/PSD-95 complex formation, signaling, and facilitate the induction of LTP in AS mice. We propose that the failure of TrkB receptor signaling at synapses in AS is directly linked to elevated levels of Arc associated with PSD-95 and PSD-95 PDZ-ligands may represent a promising approach to reverse cognitive dysfunction.

## Abstract 2

Brain-derived neurotrophic factor signaling is defective in Angelman syndrome and can be rescued by disruption of Arc/PSD95 binding.

## Author Summary

Angelman syndrome (AS) is a debilitating neurological disorder caused by a dysfunctional *Ube3A* gene. Most children with AS exhibit developmental delay, movement disorders, speech impairment, and often autistic features. The Ube3A enzyme normally regulates the degradation of the synaptic protein Arc, and in its absence the resulting elevated levels of Arc weaken synaptic contacts, making it difficult to generate long-term potentiation (LTP) and to process and store memory. In this study, we show that increased levels of Arc disrupt brain-derived neurotrophic factor (BDNF) signaling through the TrkB receptor (which is important for both the induction and maintenance of LTP). We find that the association of the postsynaptic density protein PSD-95 with TrkB is critical for intact BDNF signaling, and that the high levels of Arc in AS interfere with BDNF-induced recruitment of postsynaptic density protein-95 (PSD-95) and other effectors to TrkB. By disrupting the interaction between Arc and PSD-95 with the novel cyclic peptidomimetic compound CN2097, we were able to restore BDNF signaling and improve the induction of LTP in a mouse model of AS. We propose that the disruption of TrkB receptor signaling at synapses contributes to the cognitive dysfunction that occurs in Angelman syndrome.

## Introduction

Angelman syndrome (AS) is a severe cognitive disorder caused by loss of expression of the maternally inherited allele of the *Ube3A* ubiquitin ligase gene [1],[2]. As a result of imprinting, the paternal *Ube3A* gene is silenced, such that the maternal allele is exclusively active [3],[4]. Prominent clinical characteristics include seizures, ataxia, and mental retardation [5]. A mouse model null for maternal Ube3a [6] showed impairment in long-term potentiation (LTP) and learning [6]. Biochemically, the mice exhibited dysregulation of α-calcium/calmodulin-dependent protein kinase II (CaMKII) activity [7],[8], required for certain forms of learning [9].

Ube3A ubiquitinates and degrades the immediate-early gene Arc (activity-regulated cytoskeletal-associated protein) [10], whose expression is required for LTP consolidation [11],[12] and experience-dependent plasticity [13]–[15]. Arc promotes AMPA receptor (AMPAR) internalization [16] to reduce AMPAR-mediated synaptic transmission [17], and mediates AMPAR clearance at weaker synapses [18]. Arc has been reported to associate with postsynaptic density protein-95 (PSD-95) [19], the prototypical PDZ (PSD-95/Discs large/zona occludens-1) postsynaptic protein [20],[21], known to play a key role in the endocytosis of synaptic AMPARs [22]–[24] and to regulate AMPAR incorporation at synapses [25]–[28]. The PDZ domains of PSD-95 bind the cytoplasmic tails of select NMDA and Kainate receptor subunits [29],[30] to assemble cell-signaling scaffolds [31],[32]. To investigate the function of PSD-95, we synthesized a high affinity PSD-95 PDZ-domain peptidomimetic ligand, CN2097. The design of CN2097 (R_7_-CC-YK[KTE(β-Ala)]V), incorporates a lactam ring and a β-alanine linker that form unique contacts outside the canonical PDZ binding pocket [33],[34].

In the present study, we sought to test if the deficit in LTP-induction in AS mice might be the result of defective brain-derived neurotrophic factor (BDNF) signaling. BDNF binding to the TrkB receptor has been shown to promote the induction and maintenance of LTP [35]–[39], and BDNF or TrkB deficient mice exhibit a marked reduction in LTP [40]–[42]. We report that PSD-95 association with TrkB is critical for intact BDNF signaling. In AS hippocampal slices, the BDNF-induced association of PSD-95 with TrkB was reduced compared to wild type (WT), resulting in attenuated PLCγ (CaMKII and CREB) and PI3K (Akt-mTOR) signaling, whereas MAPK (extracellular signal-regulated kinase [Erk]) signaling was intact. In AS mice the elevated association of Arc with PSD-95 is shown to interfere with the recruitment of PSD-95 to TrkB. Treatment of AS hippocampal slices with CN2097, the PSD-95 PDZ-domain peptidomimetic ligand, increases the association of PSD-95 with TrkB to restore PLCγ and PI3K-Akt-mTOR signaling in AS mice, and to facilitate LTP. Together, these data suggest drugs based on enhancing Trk-PSD-95 interactions, such as CN2097, may provide a novel approach for the treatment of AS and autism spectrum disorders.

## Results

### CN2097, a PSD-95 PDZ Peptidomimetic Ligand, Promotes LTP in Angelman Mice

PSD-95 has been shown to regulate synaptic strength and is proposed play a key role in LTP [25],[26],[43]–[45]. Previously, we developed a bridged cyclic peptide (CN2097; Figure S1A), that binds with high affinity to the PDZ1-PDZ2 domains of PSD-95 [33],[34], and incorporates a polyarginine peptide to enable uptake by neurons (Figure S1B). To examine the effects of CN2097 on LTP, field excitatory postsynaptic potentials (fEPSPs) were elicited from hippocampal slices of AS and WT littermate male mice, 2–4 mo of age, by stimulating Schaffer collaterals and recording from the stratum radiatum of the CA1 area. LTP was considered successful when the average EPSP slope showed an increase of at least 20% lasting 55–60 min after induction. In WT mice, we found that CN2097 significantly increased the LTP induction rate under subthreshold conditions.

**Figure 1:**
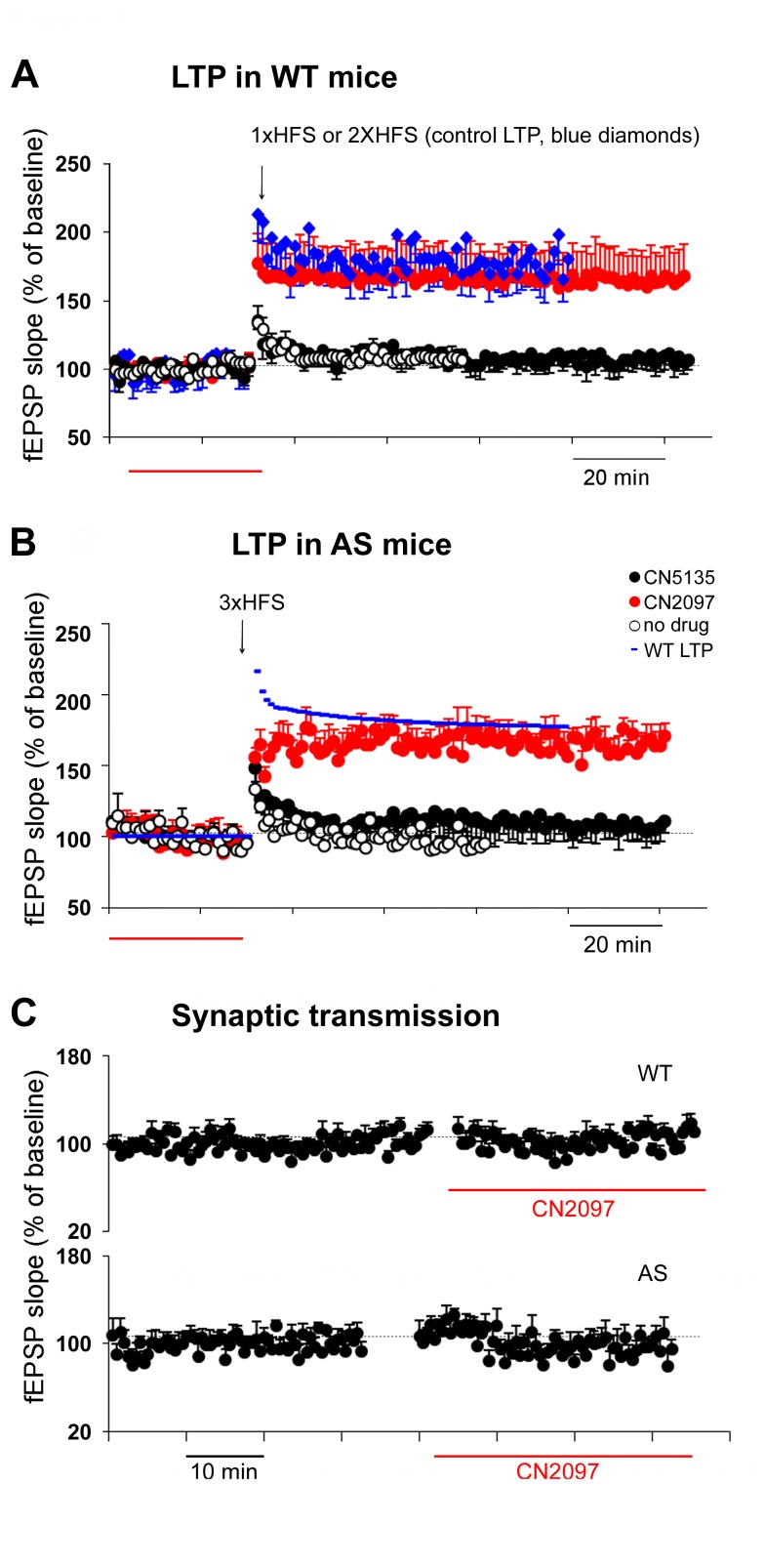
Defective BDNF-mediated synaptic plasticity in AS mice is mitigated by CN2097. Field EPSP slopes plotted as a percentage of pre-tetanus baseline in the CA1 region of the hippocampus. (A) LTP in WT mice. A single HFS train (1× HFS; 1-s train at 100 Hz) was insufficient to induce LTP (open circles), whereas 2-× 1-s trains at 100 Hz separated by 15-s resulted in LTP (blue diamonds; 2× HFS). Following application of 2 µM CN2097 for 30 min, a 1× HFS stimulus now produced significant LTP (red dots, CN2097) comparable to the 2× HFS protocol (blue diamonds). A single HFS in the presence of the negative control CN5135 had no effect on LTP (black dots, CN5135). Red bar, time of drug application. (B) LTP in AS mice. Three HFS trains 10 s apart (3× HFS) had no significant effect on fEPSP slopes (open circles, no drug). Following application of 2 µM CN2097 for 30 min, the 3× HFS stimulation protocol resulted in LTP (red dots, CN2097). No such effect was observed when the slices were incubated with the control peptide CN5135 (black dots). For comparison, WT LTP induced using the 2× HFS is shown (blue line, same data as in [A]; fitted using IGOR Pro program). Red bar, time of drug application. (C) CN2097 (2 µM, red bar) had no significant effect on baseline synaptic transmission either in WT (top) or AS mice (bottom).

Thus, a single high frequency stimulation (1× HFS, 1 s at 100 Hz) did not induce LTP in the majority of WT slices tested; with a mean fEPSP slope as a percentage of baseline of 107.9±4.8% (*n* = 11 of 14; Figure 1A). In contrast, applying two HFS trains (2× HFS; 2×1 s at 100 Hz separated by 15 s) significantly increased the induction rate of LTP (mean fEPSP slope of 178.5±15.8%, *n* = 6 of 10, *p*<0.001; Figure 1A). Importantly, in the presence of CN2097 (2 µM), the LTP induction rate of a single HFS was significantly increased, resulting in LTP in 69% of the slices tested with a mean fEPSP slope of 164.8±25.0% (*n* = 11 of 16, *p*<0.05 compared to control 1× HFS; Figure 1A). This LTP was shown to be NMDA receptor dependent, as it was blocked by APV, a competitive NMDA receptor antagonist (*n* = 8, unpublished data). CN2097 alone did not significantly affect baseline synaptic transmission (% fEPSP slope: baseline 100±0.002, CN2097 102.5±2.66, *n* = 7, *p* = 0.19; Figure 1C, top). CN5135, a negative control compound where the 0 and −2 ring positions of CN2097 were substituted with alanine residues to disrupt PDZ binding (Figure S1A), did not significantly increase the LTP induction rate of a single HFS (108.1±5.0%, *n* = 6 of 8, *p* = 0.5). These results suggest that in WT littermates of AS mice CN2097 can reduce the threshold for LTP induction.

In AS hippocampal slices, deficits in LTP were reported to be due to an alteration in the induction threshold, and LTP could be rescued by increasing synaptic stimulation at 32°C [8]. Consistent with previous studies [6]–[8],[15], we were unable to induce LTP in AS mice, using either a single (*n* = 5) or two sets of HFS (*n* = 7). Similarly, applying three sets of HFS also did not induce LTP when recorded at 30°C (3× HFS: 97±4.6%, *n* = 8 of 10; Figure 1B). However, in the presence of CN2097 (for 30 min before LTP induction) the 3× HFS protocol significantly increased the LTP induction rate, with LTP being observed in 67% of AS slices recorded with a mean fEPSP slope of 167.4±7% (*n* = 8 of 12, *p*<0.0001; Figure 1B). CN2097 had no significant effect on fEPSP slopes in AS slices (% fEPSP slope: baseline 100±0.005, CN2097 100.8±5.39, *n* = 5, *p* = 0.45, Figure 1C, bottom) of evoked synaptic responses.

### AS Mice Exhibit Deficits in BDNF Signaling

The defects in LTP induction observed in AS mice are reminiscent of those observed in BDNF and TrkB mutant mice [40]–[42],[46], which led to examine whether BDNF signaling was compromised in AS mice. NMDA receptor activity promotes the release of BDNF [47] to stimulate TrkB-PLCγ-CaMKII/CaMKIV-CREB and PI3K-Akt signaling pathways [48],[49]. BDNF-TrkB signaling increases the delivery of PSD-95 [50] and AMPAR subunits to synapses [51],[52], and is reported to play a role in transsynaptic coordination [53],[54].

**Figure 2:**
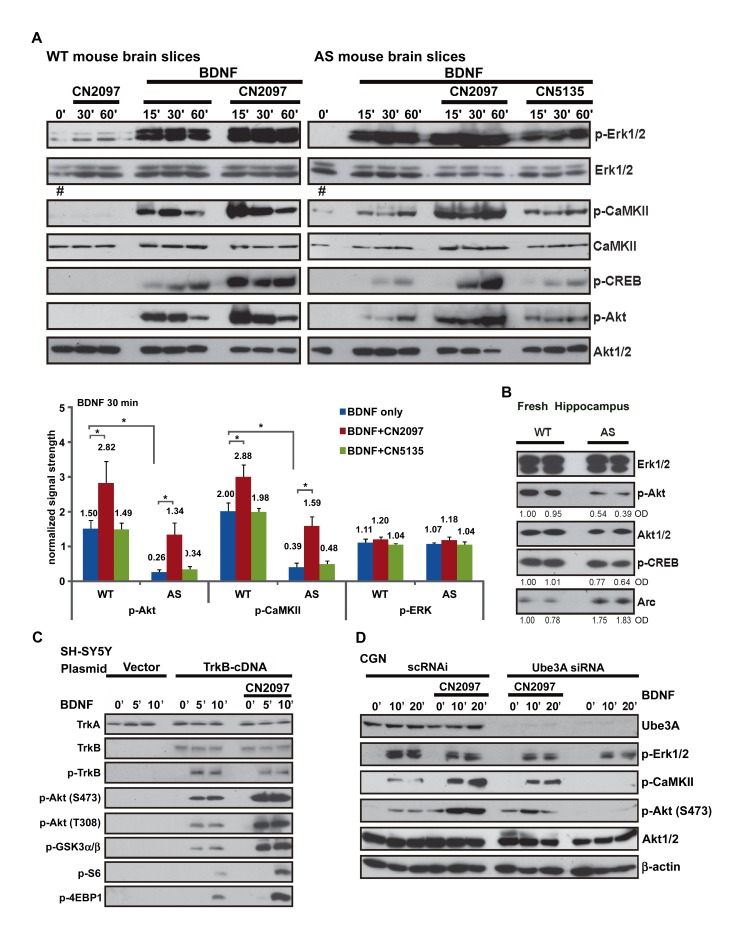
Defective BDNF signaling in AS mice and rescue with CN2097. (A) CN2097 enhances defective BDNF induced CaMKII/CREB and Akt phosphorylation in AS brain slices. Upper panel: Representative Western blots showing the relative abundance of phosphorylated protein (p-Erk1/2, p-CaMKII, p-CREB, and p-Akt-S473) from lysates prepared from WT and AS brain slices. Slices were stimulated with BDNF (50 ng/ml) in the presence or absence of CN2097 or the control compound CN5135 (2 µM; 30-min pretreatment). Slices were evaluated at designated times (‘, min) following BDNF treatment. The level of basal p-CamKIIα (phospho-Thr286) is enhanced in AS untreated slices compared to WT (0′, lanes indicated with a #). Total Erk1/2, Akt1/2, and CaMKII loading controls are shown. Lower panel: Quantitative analyses of p-Akt, p-CaMKII, and p-Erk after 30 min BDNF treatment, normalized to individual loading controls. Statistical significance was calculated using a two-tailed Student's *t* test. Data are mean ± SEM from at least three independent experiments. *p<0.05. (B) Freshly dissected AS mouse hippocampus show lower endogenous p-Akt and p-CREB expression and more Arc expression versus WT mouse. Representative Western blots showing the relative abundance of phosphorylated protein (p-CREB and p-Akt-S473) in lysates. Erk1/2, Akt1/2, and Arc are shown. Optical densities normalized to WT (left lane) are indicated below selected rows. (C) CN2097 enhances BDNF-induced PI3K/Akt/mTORC1, but not Erk signaling in SH-SY5Y cells. SH-SY5Y cells transfected with control empty vector (Vector) or TrkB cDNA (48 h) were treated with BDNF (25 ng/ml) in the presence or absence of CN2097 (2 µM, 20-min pretreatment) for the indicated time points. Phosphorylation of Akt, GSK, S6 (p70S6K), 4E-BP1, and TrkB were detected using indicated antibodies. Total, TrkA, and TrkB are shown to confirm equal sample loading and equivalent levels of TrkB-cDNA expression respectively. (D) Western blot analysis showing knockdown of Ube3A in WT CGNs inhibits BDNF signaling. CGNs (CGN; age p5) transfected with scrambled control (scRNAi, 200 nM, 24 h) or Ube3A siRNA oligonucleotides (200 nM, 24 h) were serum starved and treated with BDNF (25 ng/ml) in the presence or absence of CN2097 (2 µM, 20-min pretreatment) for indicated time points. Western blots were probed for phosphorylation of Erk, CaMKIIβ and Akt; total CaMKII, Akt, and β-actin were detected as loading controls.

**Figure 3:**
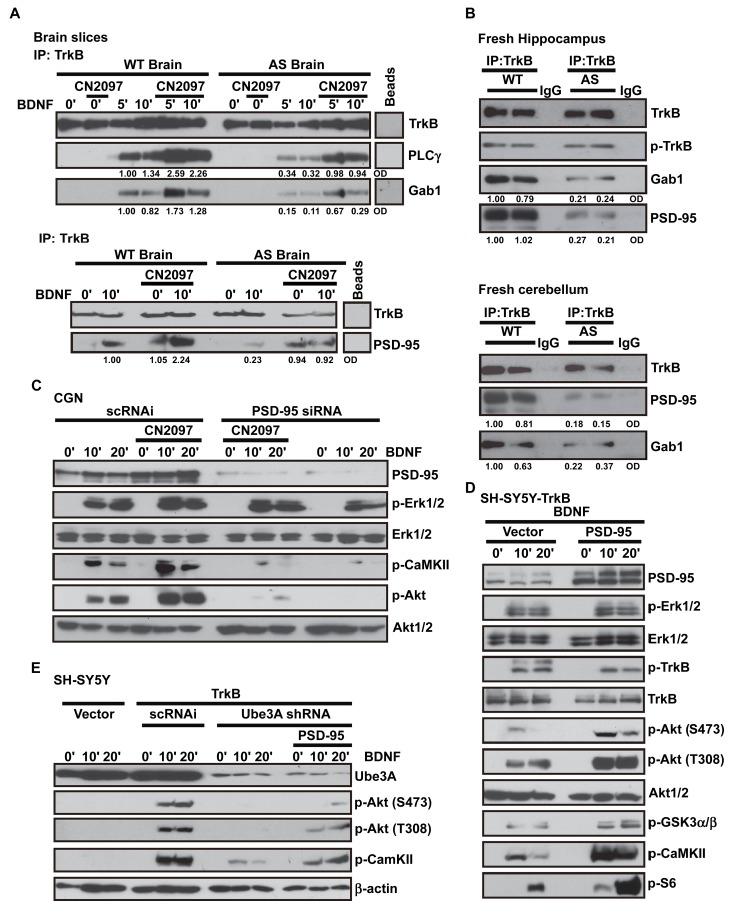
The role of PSD-95 in TrkB signaling. (A) Co-IP analysis showing the association of TrkB with PLCγ, Gab1, and PSD-95 is reduced in AS brain slices, and CN2097 restored these interactions. Representative Western blots for immunoprecipitation with an antibody to TrkB or a beads alone control (Beads), and immunoblotting with antibodies to TrkB, PLCγ, Gab1 (left upper panel) and TrkB and PSD-95 (left lower panel). Brain slices from both WT and AS littermates were pretreated with CN2097 (2 µM; for 30 min) followed by BDNF treatment (50 ng/ml). Input lanes (see Figure S3A) show the expression level of TrkB, PLCγ, and β-actin. Numbers below selected rows represent normalized optical density (OD) values. (B) Co-IP analysis showing the association of TrkB with Gab1 and PSD-95 is reduced in freshly dissected AS mouse hippocampus (upper) and cerebellum (lower). Representative Western blots for immunoprecipitation with antibodies to TrkB or IgG control, and immunoblotting with antibodies to TrkB, p-TrkB, Gab1, and PSD-95. Input lanes are shown in Figure S3B. Numbers below selected rows represent normalized OD values. (C) PSD-95 RNAi knockdown in CGNs inhibits BDNF signaling. Western blot analysis of PSD-95 expression and phosphorylation of Erk, CaMKIIβ, and Akt as a loading control. Erk1/2, generated from an identically loaded sister gel, is also shown. Primary mouse CGNs (CGNs, p5) transfected with scrambled control (scRNAi, 200 nM, 24 h) or PSD-95 siRNA oligonucleotides (sh95A; 200 nM, 24 h) were serum starved and treated with BDNF (25 ng/ml) in the presence or absence of CN2097 (2 µM, 20-min pretreatment). (D) Western blot analysis showing that overexpression of PSD-95 enhances BDNF-induced p-Akt and p-CaMKII, but not p-Erk or p-TrkB in SH-SY5Y-TrkB transfected cells. SH-SY5Y cells were transfected with TrkB+control empty vector or TrkB+PSD-95 cDNA for 48 h. Cells were treated with BDNF (25 ng/ml). Results show that increased levels of PSD-95 enhance BDNF-signaling of p-Akt (S473 and T308), p-CaMKII, pGSK3α/β, and p-S6. Akt1/2 and p-Akt (S473) were generated from an identically loaded sister gel. Akt1/2 and Erk1/2 demonstrate equal sample loading. Quantitation showing p-CaMKII and p-Akt but not p-Erk were significantly enhanced is shown in Figure S3D. (E) Western blot analysis showing that overexpression of PSD-95 restores BDNF signaling in Ube3A RNAi knockdown SH-SY5Y cells. SH-SY5Y cells transfected (48 h) with vector alone, TrkB+scramble shRNA (sc-RNAi), TrkB+Ube3A shRNA, TrkB+Ube3A shRNA+PSD-95, were treated with BDNF (25 ng/ml) for indicated time points. Western blots were probed with antibodies to Ube3A, p-Akt (S473 and T308), p-CaMKII, and the loading control, β-actin. p-Akt and p-CaMKII were generated from an identically loaded sister gel. Quantitation showing that overexpression of PSD-95 enhanced both p-Akt (*p*<0.01) and p-CaMKII (*p*<0.01) is shown in Figure S3E.

**Figure 4:**
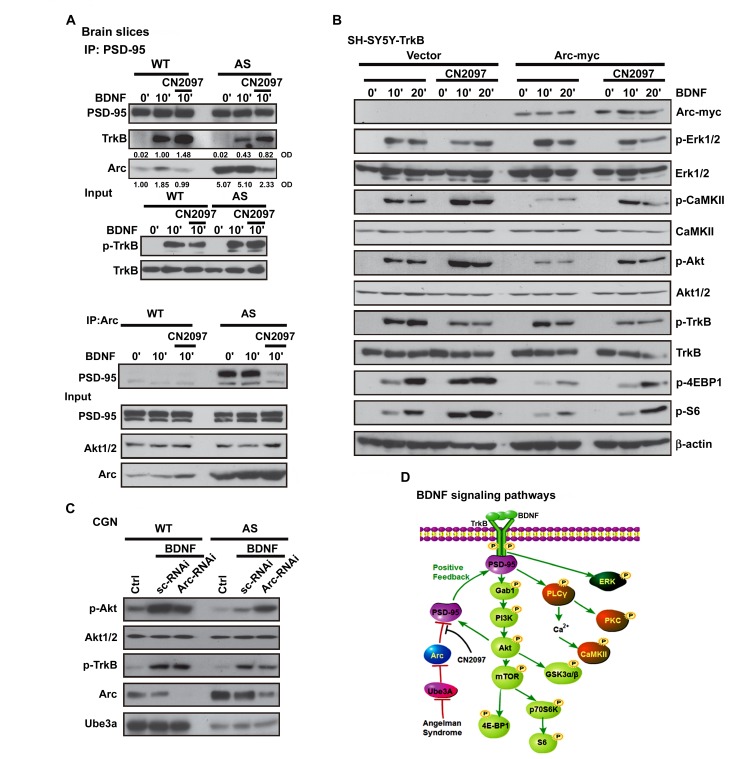
Arc interacts with PSD-95 to inhibit TrkB signaling, while CN2097 disrupts the Arc/PSD-95 association to restore signaling. (A) Upper panel: Co-IP with an antibody to PSD-95, and immunoblotting for Arc, show increased of Arc/PSD-95 association in AS brain slices compared to WT (*p*<0.05). AS slices pretreated with CN2097 significantly reduced Arc association with PSD-95 (*p*>0.1) to levels approaching WT. Numbers below selected rows represent normalized optical density (OD) values. See quantitation in Figure S4A. BDNF-induced association of PSD-95 with TrkB was diminished in AS compared to WT and was significantly increased in the presence of CN2097 (10′, *p*<0.05). Sample input was normalized to equal levels of TrkB as shown. Lower panel: Co-IP with an antibody to Arc and immunoblotting for PSD-95, show that Arc association with PSD-95 is elevated in the AS brain slices compared to WT, and that this association is not influenced by BDNF. However, pretreatment with CN2097 reduced PSD-95 association with Arc to levels approaching WT. The expression levels of PSD-95, Akt, and Arc were detected from each lysate as input control. Upper and lower panel: Coronal brain slices (hippocampus and cortex) after BDNF (50 ng/ml, 10 min) with or without CN2097 (2 µM, 30-min pretreatment). (B) Arc inhibits BDNF-induced signaling. Western blot showing that expression of c-myc-tagged Arc inhibits BDNF-induced p-CaMKII, p-Akt, p-4EBP1, p-S6 (p70S6K), but not p-Erk or p-TrkB signaling in SH-SY5Y, and CN2097 rescues. SH-SY5Y cells were transfected with empty vector control+TrkB or TrkB+Arc-myc cDNA for 48 h were treated with BDNF (25 ng/ml) with or without CN2097 (2 µM, 20-min pretreatment). The phosphorylation levels of Akt, CaMKII, 4E-BP1, S6, Erk, and TrkB and their loading controls were detected. CaMKII, p-CaMKII, and TrkB blots were generated from an identically loaded sister gel. Quantitation showing that Arc expression results in significantly lower p-Akt and p-CaMKII (*p*<0.05), which is restored to control levels by CN2097 (p-Akt, *p*>0.1; p-CaMKII, *p*>0.1) is shown in Figure S4B (lower panel). (C) Knockdown of Arc rescues BDNF signaling in AS CGN cultures. Representative Western blots probed for phosphorylation of Akt and TrkB; total Akt, Arc, and Ube3A (generated from an identically loaded sister gel) were detected. in lysates prepared from primary cultures of CGNs, derived from WT and AS mice at postnatal day 6. CGNs transfected with scrambled control (scRNAi, 200 nM, 24 h) or Arc siRNA oligonucleotides (100 nM, 24 h), at p5, were serum starved and treated with BDNF (25 ng/ml; 10′). (D) Possible role of PSD-95 in TrkB-signaling pathways. In response to TrkB stimulation, PSD-95 is recruited to TrkB, resulting in enhanced PI3K-Akt-mTOR and PLCγ1 signaling, whereas ERK activation is independent of PSD-95. The activation of the PI3K-Akt and the PLCγ1/protein kinase C (PKC) pathways may feedback and enhance the synaptic delivery of PSD-95 and TrkB signaling [50],[72]. In AS, loss of Ube3A expression results in increased levels of Arc, which associate with PSD-95 to inhibit PSD-95 recruitment to TrkB. CN2097 binds to PSD-95 to disrupt Arc association and rescues signaling.

To determine whether AS mice exhibit defects in BDNF signaling, we performed western blot analysis probed with phospho-specific antibodies to assay Erk, Akt, and CaMKII activity, in age-matched WT- and AS-coronal brain slices comprising the hippocampus and cortex [36]. In terms of Erk signaling, both WT and AS slices responded similarly (Figure 2A), with phosphorylated forms of Erk (p-Erk1/2) being significantly elevated within 15 min following the application of BDNF (25 ng ml^−1^), and remaining elevated for at least 60 min (AS and WT; *n* = 4). These results suggest that BDNF-induced Erk signaling is normal in AS mice, and infer that TrkB receptor activation is intact. Confirming that TrkB activation is unaffected, we saw no significant difference in the level of TrkB expression or TrkB phosphorylation in lysates prepared from freshly isolated WT and AS hippocampal tissue (Figure 3B), or hippocampal slices (*p*>0.05; Figure 4A, input blot). In contrast to undetectable changes in BDNF-induced Erk activation in the AS-mouse, severe signaling defects were observed in both the PI3K and PLCγ1 downstream signaling cascades (Figure 2A). As a measure of BDNF-induced PI3K activity, we examined the phosphorylation state of its downstream effector, the serine/threonine kinase Akt [49]. The level of p-Akt (Ser473) in AS mice, at 30 min post-BDNF, was 18%±4.9% of WT (*p*<0.001, *n* = 4; Figure 2A, top panel, and quantitative analysis, lower panel). Similarly, peak BDNF-induced phosphorylation of CaMKIIα (Thr286) and CREB (Ser133) was lower, reaching levels of only 14%±3.5% and 41%±5.5%, respectively, compared to WT (*p*<0.001, *n* = 4; Figure 2A, top panel, and quantitative analysis, lower panel). As reported previously [8], we found that the level of basal p-CaMKIIα (Thr286) in AS mice was greater than WT (201%±22% at 0′, # in Figures 2A and S2A), while total CaMKII remained unchanged (Figure 2A). To confirm that the reduced signaling was not a result of slice preparation or the incubation period, we repeated the experiments using lysates made from freshly dissected AS hippocampus, and found that basal p-Akt and p-CREB were similarly diminished (*p*<0.01, *n* = 3; Figure 2B). It is also worth noting that basal levels of Arc in AS mice were approximately 2-fold higher than in WT (224%±27%, *p*<0.01; Figure 2B).

As CN2097 facilitated LTP in AS and WT mice, we examined whether CN2097 altered BDNF-TrkB signaling. In both WT and AS brain slices CN2097 increased BDNF-induced downstream signaling of PLCγ1 (p-CaMKIIα and p-CREB) and PI3K (p-Akt) pathways, but did not alter Erk phosphorylation (Figure 2A, top panel, and quantitative analysis, lower panel). Specifically, in WT slices, CN2097 elicited increases in BDNF-induced p-CaMKII of 55%±13.5%, (30′, *p*<0.01, *n* = 4) and p-Akt of 93%±32%, (30′, *p*<0.05, *n* = 4), whereas the control compound CN5135 had no effect (*p*>0.1, *n* = 4; Figures 2A and S2A), demonstrating the specificity of CN2097. Significantly, in AS-slices, CN2097 increased p-CaMKII (86%±11% at 30′, *p* = 0.21, *n* = 4) and p-Akt (89%±22% at 30′, *p* = 0.35, *n* = 4) to levels comparable to WT slices (Figure 2A). In the absence of BDNF stimulation CN2097 had no effect in both WT and AS brain slices (Figures 2 and S2A).

These results suggest that CN2097 acts downstream of TrkB to facilitate activation of the PI3K and PLCγ pathways. However, in brain slices we cannot rule out that CN2097 enhances synaptic activity to indirectly potentiate TrkB signaling [55]. To study TrkB signaling in isolation of synaptic influences, we heterologously expressed TrkB in SH-SY5Y neuroblastoma cells (SH-SY5Y-TrkB). Although this cell line does not express endogenous TrkB, it expresses PSD-95, Ube3A, and Arc, making it a useful in vitro system to explore the role of these proteins in BDNF signaling. Upon BDNF stimulation of SH-SY5Y-TrkB cells, CN2097 enhanced phosphorylation of Akt (Ser473 and Thr308), GSK3α/β, and the mTORC1 downstream targets 4E-BP1 and ribosomal protein p70S6K (Figure 2C; *n* = 5), demonstrating that CN2097 directly targets downstream TrkB signaling.

The observed deficits in BDNF signaling in *Ube3A* knockout mice could also reflect indirect or developmental abnormalities. To directly assess whether Ube3A influences TrkB signaling, we used RNAi to acutely deplete Ube3A in primary cultures of mouse cerebellar granule neurons (CGN), where BDNF-TrkB signaling is known to play a critical role in CGN development and survival [56]–[58]. Recapitulating the AS hippocampal slice results, acute knockdown of Ube3A in WT CGNs (to 18%±10% of scrambled RNAi protein levels), had no effect on p-Erk signaling but severely reduced BDNF-mediated p-CaMKIIβ the isoform of CaMKII that is highly expressed in mouse CG neurons [59],[60], and p-Akt (S473) signaling (*p*<0.005; Figure 2D). Similar to the responses documented in the AS slice studies shown above, p-Akt and p-CaMKII levels were restored to WT levels with CN2097 pretreatment (Figure 2D; *p*>0.1). In addition, knockdown of Ube3A in SH-SY5Y-TrkB cells (to 10%±3.0% of untransfected protein levels), produced identical deficits in BDNF signaling (Figure S2B, upper panel, and quantitative analysis, lower right panel), and enhanced expression of Arc, a previously identified Ube3A substrate (Figure S2B, middle right panel) [10]. The specificity of the RNAi was demonstrated by coexpression with recombinant Ube3A, which rescued signaling (Figure S2C, upper panel).

Defective recruitment of signaling intermediates to TrkB could explain the deficits in Akt and CaMKII signaling observed in AS mice [49]. Using coimmunoprecipitation (co-IP) studies, we examined the association of Grb2-associated binder 1 (Gab1), a major adaptor protein required for BDNF activation of the PI3K-Akt cascade [48],[49], and PSD-95, which has been shown to coimmunoprecipitate with TrkB [50],[61]. In contrast to WT brain slices where BDNF increased PLCγ, Gab1 (Figure 3A, upper panel) and PSD-95 (Figure 3A, lower panel, and Figure 4A, upper panel) recruitment to TrkB, these interactions were diminished in AS slices (PLCγ, 28%±4%; Gab1, 23%±5.5%; and PSD-95, 15%±4%; *p*<0.001 compared to WT; Figures 3A and 4A). CN2097 improved the association of PLCγ (71%±4%), Gab1 (62%±14%), and PSD-95 (53%±9%), with TrkB in AS slices, and enhanced these interactions in WT slices (∼50%, *p*<0.001; Figures 3A and 4A). Consistent with these results, co-IP studies performed on freshly dissected hippocampal and cerebellar AS tissue also showed reduced association of PSD-95 and Gab1 with TrkB (*p*<0.01; Figure 3B). The similar deficits in both the hippocampus and cerebellum are consistent with a lack of Ube3A expression in these brain regions [62],[63]. Overall, the observed deficits in TrkB signaling in AS mice are consistent with the inability of TrkB to associate with specific signaling adaptors (Figure 4D).

### PSD-95 Is Required for BDNF Signaling

We next tested whether the presence of PSD-95 is required for BDNF-induced PLCγ1 and PI3K signaling. RNAi knockdown of PSD-95 using a synthetic siRNA duplex (sh95A) was highly effective in primary CGN cultures (18.7%±8.9% compared to WT), and significantly reduced BDNF-induced phosphorylation of Akt and CaMKII (*p*<0.01; Figure 3C), identifying a unique role for PSD-95 in neurotrophin signaling. In contrast, BDNF-induced Erk signaling was normal, inferring that PSD-95 knockdown did not affect TrkB receptor activation. In support of this, PSD-95 knockdown did not alter the levels of TrkB expression or activation in CGNs or SH-SY5Y cells (Figure S3C). PSD-95 knockdown in SH-SY5Y-TrkB cells also blocked Akt and CaMKII, but not Erk activation (Figure S3C). The specificity of the PSD-95 RNAi was demonstrated by coexpressing RNAi resistant PSD-95 along with a pRS plasmid containing the shRNA to PSD-95 (sh95A) in SH-SY5Y-TrkB cells, which restored Akt signaling (Figure S3F). Further supporting that the signaling defects are a result of reduced PSD-95 expression, a second previously characterized PSD-95 shRNA construct (sh95B) [44], similarly decreased Akt and S6 activation (Figure S3F). Importantly, the knockdown of PSD-95 abrogated CN2097 rescue of p-Akt and p-CaMKII signaling (*p*<0.001; Figures 3C and S3C), lending support to the hypothesis that the mechanism by which CN2097 rescues signaling is through its interaction with PSD-95.

If PSD-95 acts as an intermediate in TrkB activation of PLCγ1 and PI3K signaling, then increasing PSD-95 levels would be predicted to enhance BDNF signaling. The exogenous expression of PSD-95 in neurons could generate ambiguous results, as PSD-95 promotes synaptic maturation [26], which could indirectly potentiate TrkB signaling [55]. To examine whether increased levels of PSD-95 enhance TrkB signaling, we again utilized the simplified SH-SY5Y cell line system, where TrkB and PSD-95 were exogenously expressed. We found that overexpressing PSD-95 in SH-SY5Y-TrkB cells increased BDNF-induced phosphorylation of Akt (S473, T308), GSK3β, CaMKIIα (T286), and the mTORC1 downstream target p70S6K, while leaving p-Erk and p-TrkB unaffected (Figures 3D and S3D for quantitative analysis). Thus, increasing the levels of PSD-95 mimic the effect of CN2097 on BDNF signaling, leading to the hypothesis that even though PSD-95 expression in AS mice is similar to WT, PSD-95 function is compromised. All indications from this study are that CN2097 increases the amount of PSD-95 available to bind to TrkB, which in turn facilitates signaling. Extending this rational, increasing the levels of PSD-95 would be predicted to overcome the effects of Ube3A knockdown. Testing this hypothesis, we found that overexpression of PSD-95 in SH-SY5Y-TrkB cells, in which Ube3A was knocked down, partially restored BDNF-induced Akt (S473 and T308) and CaMKII phosphorylation (Figures 3E and S3E for quantitative analysis). Co-transfection with other related MAGUKS (SAP97, SAP102, and Chapsyn110) did not facilitate TrkB-induced p-Akt or p-S6 signaling (Figure S3G), which is consistent with the higher PDZ binding affinity of CN2097 for PSD-95 and not these MAGUKs [33]. We also would anticipate that if CN2097 is specific for PSD-95, it should not affect signaling pathways that do not involve PSD-95. In agreement, neither CN2097 nor PSD-95 knockdown had any effect on altering insulin-like growth factor-1 (IGF) (Figure S3H) or epidermal growth factor induced signaling (unpublished data). Overall, these results show that PSD-95 plays a key role in BDNF-induced PLCγ1 and PI3K signaling, and suggest that in Ube3A-deficient neurons the association of PSD-95 with TrkB is decreased, resulting in defective signaling.

### Elevated Levels of Arc Attenuate BDNF-TrkB Signaling

Loss of Ube3A expression in AS mice result in increased levels of Arc (Figures 2B and 4A, lower panel), which has been shown to deleteriously affect synaptic plasticity [10],[16]. Arc has been reported to bind the SH3-GK domain of PSD-95 [64], and proteomic studies show that Arc is one of the proteins to co-purify with PSD-95 [19], raising the possibility that Arc's association with PSD-95 influences BDNF signaling. Co-IP studies performed from hippocampal slices are consistent with this hypothesis, showing that higher amounts of Arc co-IP with PSD-95 in AS compared to WT slices (Figures 4A, upper blot, and S4A, upper panel for quantification). Conversely, higher amounts of PSD-95 co-IP with Arc in AS compared to WT slices (Figures 4A, lower panel, and S4A, lower panel showing co-IP performed with normalized Arc levels). Interestingly, Arc appears to associate with PSD-95 independently of BDNF stimulation (Figure 4A), which was also observed in SH-SY5Y-TrkB cells co-expressing Arc-myc (Figure S4B, upper and quantitation in middle panel). Treatment of the brain slices with CN2097 reduced the Arc-PSD-95 interaction, which was coincident with increased association of TrkB receptors with PSD-95 (Figure 4A).

To directly test the hypothesis that Arc expression can interfere with BDNF signaling, we expressed Arc in SH-SY5Y-TrkB cells and examined TrkB signaling. Arc overexpression decreased the levels of BDNF-induced p-Akt, p-CaMKII, p-4EBP1, p-S6 (Figures 4B and S4B, lower panel quantitation), but not p-Erk or p-TrkB, identical to knockdown of PSD-95 (Figure 3C). Significantly, CN2097 restored the defective CaMKII, Akt, 4EBP1, and S6 signaling resulting from Arc overexpression to control levels (Figures 4B and S4B, lower panel quantitation), and disrupted the interaction between Arc and PSD-95, with a concomitant increase in BDNF-induced TrkB-PSD-95 association (Figure S4B, middle panel). In a different approach to increase Arc levels we expressed a dominant negative Ube3A (E6AP C833A) [65] in the SH-SY5Y-TrkB cells. We also observed significantly reduced BDNF-induced p-Akt signaling when compared to SH-SY5Y-TrkB control cells (Figure S4C), which was rescued with an Arc-shRNA (Figure S4C) [10].

If Arc is a critical inhibitor of PSD-95-TrkB interaction and downstream signaling in AS mice, then decreasing Arc expression in AS neurons would be predicted to rescue TrkB downstream signaling. Using a previously described Arc siRNA [10], we knocked down Arc in primary cultures of CGNs prepared from the cerebellum of individual postnatal (day 5) WT and AS mice. Ube3A expression is extremely low in the cerebellum of AS mice (Figure S3B), and as shown in Figure 4C, AS granule neuron cultures had greatly reduced expression of Ube3A compared to WT, resulting in increased expression of Arc. These AS cultures exhibited reduced BDNF-induced Akt signaling (comparing sc-RNAi transfected WT and AS, *p*<0.01; Figure 4C), consistent with the Arc overexpression studies performed in SH-SY5Y-TrkB cells (Figure 4B). Arc-RNAi reduced Arc expression, and in AS neurons restored BDNF-induced Akt phosphorylation to WT control levels (*p*>0.1). We also observed that knockdown of Arc in WT neurons decreased BDNF-induced Akt signaling (Figure 4C; *p*<0.01), suggesting that basal levels of Arc are needed for TrkB signaling. This observation is not unexpected as the expression of Arc appears to be finely tuned to regulate LTP [11],[12],[66], endocytosis [16], and LTD [12],[17].

### AS Mice Exhibit Deficits in PSD-95 and Synaptophysin Synaptic Localization

TrkB signaling has been reported to be required for synaptic localization of PSD-95 [50] and excitatory synapse formation [67]. This finding, together with the observation that AS mice exhibit abnormal synapse formation in the CA1 region of the hippocampus [62], suggests that the synaptic localization of PSD-95 may be reduced in AS neurons. To investigate this we performed a quantitative analysis of synaptic puncta density in the hippocampal CA1, CA3, and dentate gyrus (DG) subregions, using antibodies to synaptophysin (SYN) and PSD-95 to visualize presynaptic and postsynaptic specializations, respectively.

**Figure 5:**
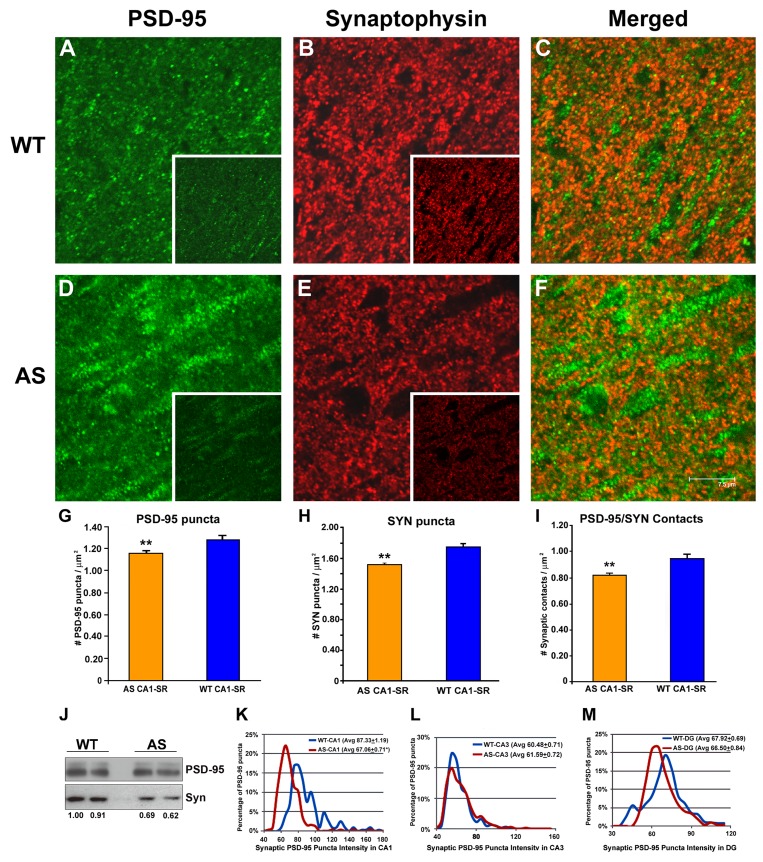
Decreased synaptic density in the CA1 hippocampal area. PSD-95 (green, A and D) and SYN (red, B and E) antibodies were used to stain synapses (merged, C and F) in stratum radiatum (SR) of the hippocampal area CA1. Here differences in PSD-95 and SYN staining were observed between WT and AS mice. In the case of SYN, there was a noticeable decline in staining intensity in the AS mouse (see unthresholded image in the inset of [E] compared to the unthresholded image taken from the WT mouse (see inset of [B]). Quantitative data for PSD-95 (G) and SYN (H) show a significantly reduced number of PSD-95 and SYN stained puncta in the AS mouse. Importantly, the total number of PSD-95/SYN contacts (synaptic density) was also decreased in the AS mouse (I). Western blot analysis of WT and AS hippocampus shows no visual differences in PSD-95 levels between WT and AS mice (J); however, there was roughly a 35% lower level of expression of SYN in AS than in WT. Measurement of synaptic PSD-95 puncta intensities are shown in CA1 (K), CA3 (L), and DG (M). In both CA1 (K) and to a lesser extent in the DG region (M), there was a shift to the left with respect to the number of puncta that were less intensely stained in the AS mouse as compared with WT. In contrast no differences were noted in CA3 (L). Immunostained sections were imaged on a confocal microscope using 100× objective and a 4× zoom. Scale bar = 7.5 µm. (*p<0.05).

To first examine for differences between WT and AS mice we used Image J software to compare the relative numbers and intensity of staining of discrete PSD-95 and SYN immunolabeled puncta in the CA1, CA3, and DG subregions (Figure S5). Importantly, in the CA1 stratum radiatum (CA1-SR) region, AS mice exhibited a significant reduction in the number of PSD-95-positive puncta (0.710±0.026 puncta/µm^2^, *p*<0.05, 16.2%) compared to control mice (0.847±0.055 PSD-95 puncta/µm^2^; Figure S5A), with no significant reduction in CA3 or DG regions (Figure S5E and S5I). Examination of presynaptic puncta revealed a significant reduction in the number of SYN puncta in both the CA1 (Figure S5B) and CA3 subregions of the AS mouse (Figure S5F), with the greatest reduction occurring in CA1-SR region (28.0%) (Figure S5B). However, no difference in SYN puncta was detected in DG (Figure S5J) between AS and WT mice. In addition to a decline in SYN-puncta in the AS mouse, the total puncta intensity of CA1 SYN-IR was significantly reduced in CA1-SR (Figures 5E, insert, and S5D) compared with WT (Figures 5B, insert, and S5D). A significant reduction of SYN-staining intensity was also shown in the neuropil of CA3 (Figure S5F), but not in the DG (Figure S5J). Overall, these data indicate that the CA1 region in the AS mouse has a significant reduction in both PSD-95 and SYN puncta density and intensity. Independent conformation of SYN-decline in the hippocampus of the AS mouse was demonstrated by Western blot (Figure 5J).

We next addressed whether the number of PSD-95 contacts with SYN-IR puncta (synaptic density) was affected. Although the use of image analysis software was able to show significant relative differences between AS and WT mice in the distinct regions of the hippocampus, these data were limited by the minimal thresholding capabilities of the software, whereby faintly stained puncta, particularly in the AS mouse, were not recorded. In order to get an accurate assessment of the synaptic density in CA1, manual counting the number of PSD-95 (Figure 5A and 5D), SYN (Figure 5B and 5E), and their synaptic contacts (Figure 5C and 5F) was preformed (See Methods for description of manual counting). In agreement with previous reports [67], the distribution of PSD-95-IR puncta averaged 1.29 puncta/µm^2^±0.03, and the synaptic density averaged 0.95±0.03 in the WT mouse (Figure 5G and 5I). In the AS-CA1 region, we found that there was a significant reduction in both the number of PSD-95-IR puncta (Figure 5G; 1.15 puncta/µm^2^±0.02: *p*<0.01, two-tailed *t* test, with normality tested and confirmed using the method of Kolmogorov and Smirnov), as well as the number of synaptic contacts between PSD-95 and SYN (Figure 5I; 0.82 contacts/µm^2^±0.02, *p*<0.01), which translates to a 10.9% reduction of PSD-95-IR puncta and a 13.7% decline in synaptic density in the AS-mouse within the neuropil region of CA1. These data confirm that in the AS CA1 region there is a significant reduction in the number of synapses.

Confirming that the PSD-95-IR staining was in agreement with previous reports in the WT-mouse [67], we next sought to determine if there is a shift in the distribution of staining intensity of individual PSD-95-puncta making contact with SYN in the AS-mouse. Mean intensity for each synaptic PSD-95 puncta was measured by placing a 34-pixel circle over the punctum using ImageJ (see Methods). Results showed that the mean intensity of puncta in AS CA1 was lower than WT (Figure 5K), and plotting the distribution of all measured puncta as a function of mean intensity showed that there is a shift to the left in the AS mouse, indicating that a significant number of synaptic PSD-95-IR puncta in the AS mouse are less intensely stained than in WT. Also, despite this shift to less-intensely stained synaptic puncta in the AS mouse, there was no difference noted between AS and WT CA1-SR in the average intensity distribution of all identified PSD-95 stained puncta (Figure S5C), indicating that synaptic PSD-95 puncta are preferentially compromised. A similar loss in synaptic PSD-95 staining intensity was observed in the DG region (Figure 5M) but not in the CA3 (Figure 5L). These results are consistent with deficits in TrkB signaling [50].

## Discussion

Numerous studies show that BDNF plays a critical role in synaptic plasticity and memory [68],[69]. Significantly, the deficits in synaptic plasticity observed in AS mice are similar to those in BDNF or TrkB mutant mice [40]–[42]. TrkB-PLCγ signaling on both sides of the synapse has been reported to be required for LTP induction [70], and synaptic activity is believed to trigger the secretion of BDNF either pre- [71] or post-synaptically [47], to activate postsynaptic TrkB-PI3K signaling and increase PSD-95 transport to synapses [50],[72]. Suppression of TrkB signaling leads to a reduction in the number and intensity of PSD-95 puncta along dendrites [72] and *TrkB* knock-out mice show decreased numbers of hippocampal Schaffer collateral excitatory synapses [67],[73]. Our data show that in the hippocampal CA1 region of AS mice there is a significant reduction in the number of synapses, and a decrease in the intensity of PSD-95 puncta colocalized with SYN (Figure 5I and 5K). These results are compatible with the reported abnormalities in spine morphology, number, and length in CA1 pyramidal neurons of *Ube3a* maternal-deficient mice [62], and are consistent with the impaired LTP in this region (Figure 1B), and compromised cell signaling mediated by BDNF/TrkB in CA1 pyramidal cells.

We have identified PSD-95 as a novel TrkB-associated protein, critical for full activation of downstream PI3K-Akt and PLCγ signaling. We found that BDNF-induced association of PSD-95 with TrkB is impaired in *Ube3A* m−\p+ mice, resulting in reduced activation of signaling molecules downstream of TrkB, including Akt-mTORC1 and PLCγ-CaMKII, whereas the Erk pathway appeared intact. The in vivo defects in BDNF signaling observed in AS mice were recapitulated in vitro by depletion of Ube3A or increasing the expression of Arc, while elevating the expression of PSD-95 restored defective signaling. Moreover, a bridged cyclic peptide (CN2097) shown by nuclear magnetic resonance (NMR) studies to uniquely bind the PDZ1 domain of PSD-95 with high affinity [33],[34] enhanced BDNF-induced TrkB-PSD-95 complex formation, to improve signaling in AS brain slices. Depletion studies show that PSD-95 is essential for the effectiveness of CN2097 restoration of signaling in *Ube3* deficient neurons. Rescue of BDNF signaling by CN2097 was shown to strongly correlate with the ability of CN2097 to decrease PSD-95 association with Arc. Overall, these studies demonstrate PSD-95 enhances TrkB-induced PLCγ and PI3K signaling pathways (Figure 4D), which could further facilitate PSD-95 trafficking to synapses [50] and modulate NMDA receptor-dependent synaptic plasticity [69],[74].

In addition to postsynaptic signaling deficits in the AS mouse, we also observed deficiencies in the presynaptic terminals, where immunostaining for the synaptic vesicle protein SYN was shown to be significantly less in CA1-SR in the AS mouse (Figure 5E, inset), as compared with a WT littermate (Figure 5B, inset). In agreement with the reduction of the average intensity of SYN puncta (∼53%; Figure S5D), the expression level of SYN in the hippocampus of *Ube3A* m−\p+ mice, by Western blotting, was greatly reduced (∼30%; Figure 5J), as reported previously [75]. In addition, there was a significant reduction in the number of SYN puncta in this region of the AS mouse (Figure 5H). It is possible that loss of Ube3A, which has been shown to also localize to the presynaptic compartment [62], leads to an impairment in presynaptic function. In support of this, Philpot and colleagues found a decrease in the number of synaptic vesicles at both excitatory and inhibitory synapses of Ube3a^m−/p+^ mice, resulting in a severe deficit in inhibitory drive to neocortical pyramidal neurons [76]. Alternatively, the reduction in the number of SYN puncta in AS mice could be the indirect result of defective transsynaptic BDNF signaling [53],[54]. BDNF and TrkB knockout mice also show a reduction in SYN levels in synaptosomes, leading to a reduction in docked synaptic vesicles and synaptic fatigue [46],[73],[77]. Prolonged BDNF treatment reversed the synaptic fatigue observed in BDNF knockout mice, suggesting a role for BDNF in the mobilization and/or docking of synaptic vesicles [35],[46].

Mechanistically, the specific function of PSD-95 in BDNF-mediated signaling remains to be elucidated. Increasing evidence indicates that Trk activation of differential signaling pathways is tied to their internalization and trafficking to endosomes [78],[79]. Lipid rafts also appear to be essential for Trk-activation of the PLCγ pathway [80]. Interestingly, in Fyn-deficient mice TrkB translocation to lipid rafts is prevented and mice exhibit compromised BDNF-induced PLCγ, PI3K-Akt, but not Erk signaling [80], identical to our findings in AS mice. PSD-95, which associates with lipid rafts by palmitoylation [81], and has been shown to bind Fyn [82], may be required for the intracellular sorting or retention of TrkB to lipid rafts [83].

Our data are consistent with the hypothesis that, in AS mice, the increased levels of Arc and its association with PSD-95 interfere with BDNF/TrkB-dependent recruitment of PSD-95 (Figures 3A and 4A). However, it still remains to be determined if Arc directly binds to PSD-95 or if linking proteins are required for its association. One possibility is that Arc could bind indirectly to PSD-95 via dynamin or endophilin to regulate TrkB endocytic trafficking and signaling from early endosomes. Trk signaling is dependent on internalization via a dynamin-dependent process and endophilins regulate BDNF-TrkA early endocytic trafficking and signaling [84]. Arc directly binds and recruits endophilin and dynamin to early/recycling endosomes [16], and recent studies indicate that Arc plays a role in the postsynaptic trafficking of amyloid precursor protein [85]. Furthermore, Arc and PSD-95 are both critical for AMPAR trafficking [16],[22],[25],[27],[86], raising the possibility that the association of Arc with PSD-95 may also provide a mechanism to control AMPAR trafficking.

A number of recent studies aimed at restoring Ube3A activity in the AS mouse demonstrate that it may be possible to improve learning and memory in adult AS patients [87],[88]. The discovery that CN2097 can reinstate BDNF-signaling pathways in AS mice to facilitate LTP suggests that the development of drugs targeting PSD-95 may be beneficial in treating the behavioral deficits in AS. Therapeutic strategies that enhance neurotrophin signaling may be additionally advantageous as signaling through these receptors appears to be compromised in other neurological diseases [89],[90].

## Materials and Methods

### Electrophysiological Recordings and Data Analysis

2–4-mo-old male WT and AS mutant (Ube3A^m−/p+^) mice were bred on a 129S7 background strain, supplied by Jackson laboratories (stock number 00447) [6], were deeply anesthetized with isoflurane, then decapitated. Brains were rapidly removed and placed in 4°C dissecting solution (containing 60 mM NaCl, 3 mM KCl, 1.25 mM NaH2PO_4_, 28 mM NaHCO_3_, 110 mM sucrose, 0.6 mM ascorbic acid, 5 mM Dextrose, 7 mM MgCl_2_, and 0.5 mM CaCl_2_.H_2_O, [pH 7.25–7.35]). Brains were then sectioned and glued to the stage of a vibrating blade microtome (Vibratome) and coronal “brain slices” (450 µm) containing the dorsal hippocampus and adjoining cortex were cut and incubated in a humidified interface chamber containing oxygenated artificial cerebral spinal fluid (ACSF) (95% O_2_/5% CO_2_) containing 119 mM NaCl, 2.5 mM KCl, 1 mM NaH_2_PO_4_, 26 mM NaHCO_3_, 11 mM glucose, 1.3 mM MgSO_4_.7H_2_O, and 2.5 mM CaCl_2_ with pH (7.25–7.35) at room temperature for >1–2 h prior to use. For Western blot analyses, several brain slices were transferred to room temperature 20-ml submersion chambers containing continually oxygenated ACSF. Test reagents were added directly to the chamber.

For electrophysiological recordings, slices were transferred to a 1-ml submersion-type recording chamber perfused with 30°C, oxygenated ACSF at 2 ml/min^−1^. Borosilicate glass microelectrodes (resistance <1 MΩ) were placed in CA1 stratum radiatum for extracellular recordings. Synaptic responses were elicited by stimulation of the Schaffer Collaterals with 0.3-ms square wave pulses with a concentric bipolar electrode. Stimulation intensity was adjusted to record stable (<5% drift) fEPSPs at 50% of maximum amplitudes (>2 mV minimum). fEPSPs were recorded (AxoClamp2B amplifier, Axon instruments), Bessel filtered at 1 Hz and 1 kHz (Dagan, EX1 Differential Amplifier), digitized at 10 kHz (NI BNC2010A), and stored for analysis (Igor pro, Neuromatic and nClamp, www.neuromatic.thinkrandom.com). HFS trains consisted of one, two, or three 1-s 100-Hz, 0.2-ms pulse duration, over 30 s. Effects were presented as average ± SEM. Significance was determined using paired *t* tests.

### Animals

To obtain heterozygous AS mice missing the maternally *Ube3A* ^m−/p+^ gene, we crossed a heterozygous female mouse with a WT male mouse. In all experiments we used male mice aged between 2–4 mo. Control mice were age-matched, male, WT littermates. Mice were raised on a 12-h light/dark cycle, with food and water available ad libitum and were housed in groups of two to three per cage. Electrophysiological recordings were obtained from at least three mice for each condition. All animal procedures were performed in compliance with the US Department of Health and Human Services and the IACUC animal care guidelines at Brown University.

### CGN Purification

Methods for isolation and purification of murine and rat CGNs were as previously described [91],[92]. CGNs were purified from P5 mouse pups, resuspended in serum-free medium (SFM) and plated at a density of 2.5×10^6^ cells/well, six-well plate) with poly-L-ornithine (0.01%; Sigma) and mouse laminin (20 µg/ml; Invitrogen). SFM was composed of Eagle's basal medium with Earle's salts (BME; Gibco) supplemented B27, bovine serum albumin (10 mg/ml), all from Sigma, and glutamine (2 mM; Gibco), glucose (0.5%) and penicillin/streptomycin (20 U/µl; Gibco).

### Immunohistochemistry for Synapse Labeling and Counting

Brains were fixed by perfusion of the animals under deep anesthesia (Ketamine, 75 mg/kg bw and Medetomidine, 0.5 mg/Kg bw, i.p.) through the ascending aorta with 4% (w/v) paraformaldehyde in 0.1 M sodium phosphate buffer (PBS, pH 7.2); brains were removed and immersed overnight at 4°C in the same solution. Brains were then cryoprotected by soaking in 20% (w/v) sucrose in PBS (12 h, 4°C) and 30% (w/v) sucrose in PBS (8 h, 4°C) and frozen in OCT by immersion in dry ice-cooled isopenthane. Brains were sectioned into a series of 30-µm-thick frontal or coronal slices; slices were post fixed, before the immunohistochemical staining, with the fixative solution for 2 h at 37°C. Immunohistochemistry was performed by the free-floating technique. After a 15 min treatment with 0.3% (v/v) Triton X-100 (in PBS), slices were immersed and gently shaken in a PBS-diluted (10%, v/v) horse serum (HS), containing 5% (w/v) BSA for 4–5 h at room temperature (“blocking” step). The immunostaining step was conducted by immersion and gentle shaking of the slices with the primary antibodies, diluted with blocking medium (anti-PDS-95 rabbit polyclonal antibody, Cell Signal, 1∶100 and anti-SYN mouse monoclonal antibody, SY38, Abcam, 1∶30), for 24–36 h at 4°C. Primary antibody specificity for PSD-95 and was confirmed using Western blot, After washing (10 min in blocking medium, three times), sections were incubated with secondary antibodies diluted in blocking medium, as biotinylated anti-rabbit antibody (Vector Laboratories,1∶300) and anti-mouse Texas Red-conjugated antibody (Vector Laboratories, 1∶300), for 4–5 h at room temperature. After antibody removal (blocking solution, 10 min, once; PBS, 10 min, three times), slices were then incubated with fluoroscein-conjugated avidin (Vector Laboratories, diluted 1∶500 with PBS), for 1 h at room temperature. After washing (PBS, 10 min, four times), the wet sections were placed on slides and coverslipped with anti-fluorescence fading fluid (Vectashield, Vector Laboratories). Control sections in which primary antibodies were omitted showed no labeled cells. Images of equivalent regions, 512×512 pixels, were made on a Leica TCS SP2 AOBS spectral confocal microscope using a 100×, 1.4 numerical aperture oil-immersion objective at a 4× zoom. A single optical section (0.8-µm thick) was taken from each tissue section, and at least five sections per region of interest were analyzed for each animal. All microscope settings were unchanged from section to section.

Due to the reduced intensity of PSD-95 and SYN immunostained puncta in the AS mouse, a significant number of the weaker stained opposing puncta could not be resolved using digital-quantification software; therefore, we chose to manually quantify the synaptic contacts to obtain a more accurate assessment of PSD-95/SYN contacts. PSD-95 and SYN paired confocal images were adjusted for contrast/brightness to optimize detection of the faintly stained PSD-immunostained puncta and then magnified to fit the dimensions of a high resolution flat-screen monitor (179 mm×179 mm). PSD-95-immunostained puncta were first mapped onto an acetate sheet overlay and the counted to obtain total distribution of PSD-95 puncta/µm^2^. The corresponding SYN-immunostained image was then superimposed under the PSD-95 acetate template, whereby all PSD-95/SYN contacts were then circled and quantified. Quantification of synaptic contacts was preformed blindly by at least three separate individuals. Data represent a minimum of seven sets of non-overlapping images/region/strain quantified blind. Only direct SYN/PSD-95 contacts were counted, i.e., PSD-95 puncta in apposition to SYN-immunoreactive puncta, were then assessed with Image J for their corresponding staining intensities by placement of a circle 32 pixels^2^ over each of the thresholded puncta. It is important to note that in Image J, thresholding an image does not change the value of the puncta intensity from the non-thresholded image. Groups of animals were compared using a two-tailed, two-sample equal variance *t* test.

### Plasmids and RNAi Transfection

WT human Myc-DDK-tagged Arc cloned into the pCMV6 vector was obtained from OriGene and HA-E6AP cloned into pCMV4 was from Addgene. The WT-TrkB cDNA was a gift from Luc De Vries and Myc–PSD-*95* cloned into GW1-CMV (British Biotechnology) was a gift from Morgan Sheng. Knockdown was performed using HUSH technology (Origene). A pRS plasmid vector (Origene) containing shRNA that is effective against human or mouse Ube3A (5′AGGTTACCTACATCTCATACTTGCTTAA); human and mouse PSD-95 (5′-GG AGA CAA GAT CCT GGC GGT CAA CAG TGT, sh95A; and 5′-AAT GGA GAA GGA CAT TCA GGC GCA CAA GT, sh95B) were purchased from Origene. RNA duplex oligonucleotides (RNAi) against Ube3A (5′-GUUACCUACAUCUCAUACUUGCUUUAA) or PSD-95 (5′-AGA CAA GAU CCU GGC GGU CAA CAG UGU, sh95A; and AAT GGA GAA GGA CAU CCA GGC ACA CAA GT, sh95B) were purchased from Integrated DNA Technologies (IDT). Arc shRNA was generated using the previously described sequence [10]. Plasmid transfection into neuroblastoma SH-SY5Y cells was performed with Lipofectamine 2000 (Life Technologies) in six-well plates. A pEGFP cDNA plasmid was added to determine the efficiency of transfection which was at least 30%–40% for each experiment.

For knockdown of Ube3A or PSD-95 primary CGNs, dense cultures were grown for 3 d in vitro and then switched to DMEM (no FCS) medium 1 h before transfection. 2 µl of Ube3A- or PSD-95-specific RNAi duplexes (100 µM, diluted in siRNA dilution buffer [Santa Cruz Biotechnology] and 3.0 µl of Lipofectamine PLUS Reagent [Invitrogen]) were diluted in 90 µl of siRNA dilution buffer. To this was added 2.8 µl of Lipofectamine LTX and incubated for 30 min at room temperature. The complex was added to the well containing 1 ml of DMEM for 2 h, with a final siRNA concentration of 100 nM. 10% FCS medium was then added back to the CGN and cultured for 24 h before DMEM starvation and further treatment.

### Antibodies

Antibodies against Gab1 (sc-9049), PLCγ1 (sc-166938), Arc (sc-15325), CaMKIIα (sc-13141), Erk1/2 (sc-93), and Akt1-2 (sc-8312), goat antibody against rabbit immunoglobulin G (IgG) conjugated to horseradish peroxidase (HRP) (sc-2030), and goat antibody against mouse IgG-HRP (sc-2031) were purchased from Santa Cruz Biotechnology. Mouse monoclonal antibody (mAb) against PSD-95 (clone K28/43) (MABN68) was purchased from Millipore, rabbit monoclonal antibody against PSD-95 (cs-3450) was purchased from Cell Signaling Tech. Mouse mAb aAnti-Ube3A (E6AP) (clone Ex-8) was obtained from Enzo Life Sciences (BML-PW0535). P-CaMKII (Thr286/287, clone 22B1) was obtained from Cayman Chemicals and β-actin (mouse mAb) was purchased from Sigma. P-Akt (Ser473) antibody (9271) was used unless otherwise indicated as p-Akt (Thr308) antibody (9275); p-GSK-3α/β (Ser9/21) antibody (9336), p-S6 ribosomal protein (Ser235/236) antibody (2211), p-p70 S6 Kinase (Thr398) antibody (9209), p-4E-BP1 (Ser65) antibody (9451), p-p44/42 MAPK (Erk1/2) antibody (9102), p- CREB (Ser133) antibody (9191), TrkB antibody (4606), and p-Trk (C35G9) Rabbit mAb (4619) were obtained from Cell Signaling Technology.

### Western Blotting Analysis and Immunoprecipitation

CGNs, SH-SY5Y cells or brain slices treated with the appropriate stimuli were lysed with lysis buffer (200 mM NaCl [pH 7.4], 1% Triton X-100, 10% glycerol, 0.3 mM EDTA, 0.2 mM Na_3_VO_4_, and protease inhibitor cocktail [Roche Diagnostics]). Aliquots of 40 µg of protein from each treatment were separated by 10% SDS-PAGE and transferred onto a PVDF membrane (Millipore). After blocking with 10% instant nonfat dry milk for 1 h, membranes were incubated with specific antibodies overnight at 4°C followed by incubation with secondary antibodies (HRP-conjugated anti-rabbit or anti-mouse IgG at the appropriate dilutions) for 1 h at room temperature. Antibody binding was detected with the enhanced chemiluminescence (ECL) detection system (Amersham Biosciences). Western blot results were quantified by using Image J software (NIH) after normalization to their individual loading controls. For IP, aliquots of 700 µg of proteins from each sample were precleared by incubation with 20 µl of protein A/G Sepharose (beads) (Amersham) for 1 h at 4°C. Pre-cleared samples were incubated with specific antibodies in lysis buffer overnight at 4°C. 30 µl of protein A/G beads were added and the samples were incubated for 2 h at 4°C. The beads were washed five times with phosphate-buffered saline (PBS) (4°C) and once with lysis buffer, boiled, separated by 10% SDS-PAGE, and transferred onto a PVDF membrane followed by Western blotting analysis as described above. 40 µg of protein from each treatment was also utilized for Western blot as input controls. All blots are representative of at least three independent experiments.

### CN2097 Synthesis

The peptide KNYKKTEV, was cyclized between the valine (V) and threonine (T) residues via a β-lactam alanine and linked to a seven member linear poly-arginine tail (R_7_) by a disulfide bond (CN2097: R_7_-CC- KNYKKT[βA]EV, MW2376) to enhance its diffusion and uptake capacity by neurons in intact tissues. The cyclic and poly-arginine moieties were synthesized and purified separately and subsequently coupled. A negative control peptide was prepared by introducing two disruptive alanine residues at the critical 0 and −2 ring positions that comprise the critical binding region (CN 5135). Standard fmoc-based protocols were used to synthesize all peptides. The chemical structures were determined using a high-resolution time-of-flight electrospray mass spectrometer. Reagents were sourced from Fisher, BA Chemicals, Berry, Wikem, Sigma, Novabiochem, Chempep, Quanta, and RSP amino-acids.

### Statistics

A two-tailed Student's *t* test was used to test for statistical significance in electrophysiological results and for measuring statistical significance of quantitative Western blot and imaging analyses. GraphPad Prism data analysis software was used for graph production and statistical analysis. Values are presented as mean ± SEM. Results were deemed significant where *p<0.05, **p<0.01, and ***p<0.001.

## Supporting Information

Figure S1 (A) CN2097: Design of a cell-permeable PDZ domain-targeting macrocycle. Standard Fmoc-based protocols [93], were used to synthesize the cyclic-peptide, CN2097, targeting the PDZ domain of PSD-95. The peptide, KNYKKTEV, was cyclized between the Val and Thr residues via a β-alanine linkage and linked to a poly-arginine tail to enhance its uptake by neurons. Also shown is a control cyclic peptide, CN5135, having the Ala/Ala double mutation at the 0/−2 positions, which knocks out binding to PDZ domains. (B) In situ uptake of CN2097 into rat neurons. (A) Pyramidal cells located in CA1 by 24 h following intra-ventricle injection of TMR-tagged CN2097. (B) Similar uptake was noted in multiple populations of rat retinal ganglion cells 6 h following intravitreal injection. (C) Although not as robust as shown in (B), selective uptake in retinal ganglion cells was noted 8 h following an intravenous injection of FITC-tagged CN2097, indicating that the peptide mimic is not only capable of crossing meningeal membrane barriers and selectively taken up by neurons, but also has the ability to cross the blood/brain barrier.
(TIFF)

Figure S2 (A) Western blots showing that CN2097 alone does not stimulate Erk, Akt, or CaMKII signaling in AS brain slices, and that the control compound CN5135 does not enhance BDNF signaling over levels produced by BDNF alone in WT slices. WT slices exposed to CN2097, show a 2- to 3-fold enhancement of BDNF-induced p-Akt and p-CaMKII signaling (*p*<0.01), over slices treated with BDNF alone. The level of basal p-CaMKIIα (phospho-Thr286) is enhanced in AS untreated slices compared to WT (0′, lanes indicated with an #). Blots were probed for p-Erk1/2, p-CaMKII, p-Akt-S473, and Akt loading control. WT slices were stimulated with BDNF (50 ng/ml) in the presence or absence of CN2097 (2 µM), or the control compound CN5135 (2 µM; 30-min pretreatment). AS brain slices were treated with CN2097 (2 µM). (B) CN2097 improves BDNF signaling in Ube3A knockdown SH-SY5Y-TrkB cells. Upper panel: Western blot analysis of protein lysates prepared from TrkB transfected SH-SY5Y cells (SH-SY5Y-TrkB) cotransfected with scrambled control shRNA (scRNAi) or Ube3A shRNA (48 h), treated with BDNF (25 ng/ml) in the presence or absence of CN2097 (2 µM, 20-min pretreatment). Expression of Ube3A, p-CaMKII, p-Akt, p-GSK, p-S6, and CaMKII (sister gel) are shown. Knockdown of Ube3A impeded BDNF-induced p-CaMKII, p-Akt, p-GSK, and p-S6 activation. Lower panel: Depleting Ube3A expression using Ube3A shRNA did not alter BDNF induced pErk activation compared to scrambled RNAi control. Akt1/2 and PSD-95 levels were also not affected by Ube3A knockdown and serve as loading controls. Middle right panel: Knockdown of Ube3A enhances Arc expression in SH-SY5Y cells. SH-SY5Y-TrkB cells transfected with scRNAi or Ube3A shRNA show increased Arc levels in Ube3A-depleted cells, whereas PSD-95 levels were unaffected. Lower right panel: Quantitation of the p-Akt western blot lanes (left panel). Knockdown of Ube3A results in a significant loss of p-Akt (*p*<0.05) and CN2097 restores p-Akt levels to control levels (*p*>0.1). # indicates the column that is normalized to 1.0. (C) Specificity of Ube3A RNAi. Expression of Ube3A restores BDNF signaling in SH-SY5Y cells in which endogenous Ube3A was depleted with Ube3A RNAi. Western blot analysis of protein lysates prepared from SH-SY5Y cells transfected with TrkB (SH-SY5Y-TrkB, 48 h) and the following plasmids: scrambled shRNA, termed scRNAi; Ube3A shRNA+empty vector; Ube3A shRNA (0.1 µg)+Ube3A cDNA, followed by BDNF treatment (25 ng/ml) for the indicated time points. Blots were probed with antibodies against Ube3A, p-Erk, p-Akt, Akt (sister gel), p-TrkB, and PSD-95.
(TIFF)

Figure S3 (A) The input lanes of lysate used for WT and AS slices TrkB-coIP westerns shown in Figure 3A (upper panel) show the expression level of TrkB, PLCγ, PSD-95, and Gab1. CN, CN2097. (B) The input lanes of lysate from fresh hippocampus (hippo) and cerebellum (CB) of WT and AS, used for TrkB co-IP Western blots shown in Figure 3B, show the expression level of TrkB, PSD-95, Ube3A, and Akt1/2. (C) Knockdown of PSD-95 in SH-SY5Y-TrkB cells disrupt CN2097 enhancement of BDNF signaling. Upper panel: Western blot analysis of protein lysates prepared from TrkB transfected SH-SY5Y cells (SH-SY5Y-TrkB, 48 h) cotransfected with scrambled control shRNA (scRNAi) or PSD-95 shRNA (sh95A), treated with BDNF (25 ng/ml) in the presence or absence of CN2097 (2 µM, 20-min pretreatment). The expression level of PSD-95 and phosphorylation of Erk, TrkB, Akt, 4E-BP1, and S6 were detected. TrkB was generated from an identically loaded sister gel. The RNAi mediated knockdown of PSD-95 reduced expression to 14.1%±3.5% compared to untransfected cells, and impeded BDNF-induced p-Akt, p-4E-BP1, and p-S6 induction as compared with cells transfected with scRNAi. Furthermore, CN2097 could not rescue BDNF signaling in the PSD-95 depleted cells. Lower left panel: Quantitation of Western blot data for p-Akt. CN2097 significantly enhances p-Akt signaling (**p<0.01). Knockdown of PSD-95 blocks p-Akt signaling and prevents CN2097 rescue of signaling (*p*>0.1). # indicates column that is normalized to 1.0; ns, not significant. Lower right panel: PSD-95 RNAi knockdown in CGNs does not change the levels of TrkB expression or inhibit BDNF-induced TrkB activation. Western blot analysis of PSD-95, TrkB and ERK expression, and phosphorylation of Erk and TrkB. Primary mouse CGNs (p5) transfected with scrambled control (scRNAi, 200 nM, 24 h) or PSD-95 siRNA oligonucleotides (sh95A; 200 nM, 24 h) were serum starved and treated with BDNF (25 ng/ml, 10 min). Numbers below selected rows represent normalized optical density (OD) values. (D) Quantitation for Figure 3D. Summary data for the 20-min time point showing p-CaMKII and p-Akt (T308) but not p-Erk were significantly enhanced in cells overexpressing PSD-95 compared to control SH-SY5Y-TrkB cells (*p<0.05). (E) Quantitation for Figure 3E. Knockdown of Ube3A resulted in a significant decrease in p-Akt (T308) and p-CaMKII levels (*p<0.05), while overexpression of PSD-95 enhanced both p-Akt and p-CaMKII (*p<0.05) in Ube3A knockdown cells. (F) Specificity of PSD-95 RNAi. Left panel: Specificity of sh95A. Western blot analysis of protein lysates prepared from TrkB transfected SH-SY5Y cells (SH-SY5Y-TrkB, 48 h), co-transfected with the following plasmids: PSD-95 sh95A+empty vector; PSD-95-sh95A+rat PSD-95, followed by BDNF treatment (25 ng/ml) for 20 min. PSD-95 shRNA transfected cells show a dramatic reduction in PSD-95 levels compared with control cells (0′), resulting in a very low p-Akt, but normal p-Erk signal, in response to BDNF. Transfection of RNAi resistant PSD-95 cDNA restored BDNF-induced pAkt at 20′. Blots were probed with antibodies against Ube3A and Akt as loading controls. Right panel: PSD-95 knockdown by sh95B siRNA disrupts BDNF signaling. Western blot analysis of protein lysates prepared from primary CGNs showing PSD-95 expression and BDNF-induced Akt and S6 activation. Akt1/2 was detected as a loading control. Primary rat CGNs were prepared from newborn Sprague-Dawley pups (p5), transfected with scrambled control (scRNAi, 200 nM, 24 h) or PSD-95 siRNA oligonucleotides (sh95B; 200 nM, 24 h), serum starved and treated with BDNF (25 ng/ml) for 7 and 15 min. (G) SAP97, SAP102, or Chapsyn110 do not enhance BDNF signaling in SH-SY5Y-TrkB cells. Western blot analysis of protein lysates prepared from TrkB transfected SH-SY5Y cells (SH-SY5Y-TrkB, 48 h), co-transfected with the following plasmids: GFP, SAP97, SAP102 or Chapsyn 110 (0.5 µg/ml each, 48 h), serum starved and treated with BDNF (25 ng/ml), show that none of these MAGUKS enhanced BDNF-induced pAkt or p-S6. Western blots were probed with antibodies against p-Akt and β-actin as loading control. p-Erk and p-S6 were generated from an identically loaded sister gel. (H) PSD-95 knockdown does not affect IGF signaling in WT-CGN. Western blot analysis of protein lysates prepared from WT CGN (p5), transfected with scrambled control (200 nM, 24 h) or PSD-95 siRNA oligonucleotides (sh95A; 200 nM, 24 h), serum starved and treated with IGF (25 ng/ml), show that depletion of PSD-95 does not impede IGF-induced p-Akt activation. In addition, CN2097 does not enhance IGF-p-Akt activation in CGN cells transfected with scRNAi. Ube3A and β-actin were generated from an identically loaded sister gel.
(TIFF)

Figure S4 (A) Upper panel: quantitation for the association of Arc and PSD-95 in Figure 4A. AS mice have significantly greater levels of Arc associated with PSD-95 than their WT counterparts (*p<0.05) and CN2097 disrupts this (*p<0.05). Middle panel: Arc and PSD-95 show greater association in AS mice after normalization of the input of Arc. Co-IP assay with an antibody to Arc from lysates prepared from both WT (1 mg lysate) and AS (0.33 mg lysate; note AS Arc input is 0.85 of WT). Western blots were probed with antibodies to PSD-95 show that the association of PSD-95 with Arc is significantly greater in the AS mouse (*p*<0.05). Lower panel shows the input levels of Arc, Ube3A and PSD-95 detected in lysates normalized for Arc. Numbers below selected rows represent normalized optical density (OD) values. (B) Arc associates with PSD-95 to disrupt TrkB-PSD-95 association. Upper panel: Co-IP assay with an antibody to PSD-95 from lysates prepared from TrkB transfected SH-SY5Y cells (SH-SY5Y-TrkB, 48 h) cotransfected with control empty vector or Arc-myc-tagged cDNA. Cells were untreated or stimulated with BDNF (25 ng/ml, 10 min) in the presence or absence of CN2097 (2 µM, 20-min pretreatment). Western blots were probed with antibodies to Arc, Myc, PSD-95, and TrkB. Bar graph: Quantitation of the association of Arc and PSD-95 (*n* = 3) represented in the upper panel. Arc transfection resulted in a significant increase in its association with PSD-95 (**p<0.01), and disrupted TrkB binding. CN2097 prevented Arc association with PSD-95 (**p<0.01), and restored TrkB binding to WT levels (*p*<0.1). Middle lower right panel: The input lanes for each treatment showing equal expression and loading of PSD-95, Akt, and β-actin. Lower right panel: Quantitation of Figure 4B showing that Arc expression results in significantly lower BDNF-induced p-Akt and p-CaMKII (*p*<0.05). BDNF-signaling in SH-SY5Y-TrkB cells co-transfected with either null-vector or Arc-myc containing vector (acquired from Western blots shown in Figure 4, *n* = 3). Results show that CN2097 enhances BDNF p-CaMKII and pAkt (*p<0.05). (C) Western blot analysis showing that Arc knockdown restores BDNF signaling in cells expressing a dominant negative Ube3A (E6AP C833A). SH-SY5Y-TrkB cells and SH-SY5Y-TrkB cells transfected (48 h) with a dominant negative Ube3A (E6AP C833A) [65] +/− Arc shRNA (0.1 µg), were treated with BDNF (25 ng/ml) for indicated time points. Results show that interfering with Ube3A function elevates Arc, which in turn reduces BDNF activation of p-Akt signaling when compared to SH-SY5Y-TrkB control cells (*p*<0.001; for both S473 and T308 quantitated at the 10-min time point). Suppressing Arc in the DN-Ube3A cells restored BDNF-induced p-Akt to control levels (*p*>0.1 for both S473 and T308 at the 10-min time point). Note that BDNF-signaling of p-Erk1/2 was not affected. Western blots were probed with antibodies to Arc, p-Akt (S473 and T308), Akt (sister gel), p-Erk1/2, and the loading control Erk1/2. All blots are representative of at least three independent experiments.
(TIFF)

Figure S5 PSD-95 (green) and SYN (red) antibodies were used to stain synapses in the hippocampus. PSD-95 and SYN stained puncta quantified using image J are displayed for CA1-SR (A–D), CA3 (E–H), and DG (I–L) and showing relative distributions of stained puncta and the average staining intensities of the identified puncta between WT and AS mice. Results show that in CA1-SR there is a significant decline in the relative number of PSD-95 stained puncta in the AS mouse (A), whereas no differences were recorded in CA3 (E) and in the DG (I). Similarly, there was a significant decline in the number of SYN-puncta noted in CA1 (B) and also in CA3 (F) but not in the DG (J). In comparing the average relative staining intensities of the captured puncta, there were no differences noted between WT and AS for PSD-95 for all three regions sampled (C, G, and K); however, significant differences in the average staining intensity for SYN were recorded in CA1 (D) and CA3 (H), but not in the DG (L). Immunostained sections were imaged on a confocal microscope using 100× objective and a 4× zoom. Scale bar = 7.5 µm.
(TIFF)

## Abbreviations

AMPAR: AMPA receptor
AS: Angelman syndrome
BDNF: brain-derived neurotrophic factor
CaMKII: α-calcium/calmodulin-dependent protein kinase II
CGN: cerebellar granule neuron
co-IP: coimmunoprecipitation
DG: dentate gyrus
ERK: extracellular signal-regulated kinase
fEPSP: field excitatory postsynaptic potential
Gab1: Grb2-associated binder 1
HFS: high frequency stimulation
IGF: insulin-like growth factor
LTP: long-term potentiation
PSD-95: postsynaptic density protein-95
SYN: synaptophysin
WT: wild type
