# Correction: Impairment of TrkB-PSD-95 Signaling in Angelman Syndrome

**DOI:** 10.1371/annotation/f32bc670-c9cf-4bb0-9376-cd8cfd1053c1

**Published:** 2013-03-05

**Authors:** Cong Cao, Mengia S. Rioult-Pedotti, Paolo Migani, Crystal J. Yu, Rakesh Tiwari, Keykavous Parang, Mark R. Spaller, Dennis J. Goebel, John Marshall

The following information was missing from the Funding section: This study was supported by a Postdoctoral Award (#11POST5820019) from the American Heart Association (R.T.).

