# Correction: Impairment of TrkB-PSD-95 Signaling in Angelman Syndrome

**DOI:** 10.1371/journal.pbio.1001848

**Published:** 2014-04-08

**Authors:** The PLOS Biology Staff

JM wishes to declare the following competing interest, which should have been indicated in the research article. JM owned shares in Angelus Therapeutics. However, for clarification, no materials or support were received from this company, and no agreements were in place concerning the execution or publication of this work. In addition, a US patent application entitled “Long term potentiation with cyclic-GluR6 analogs” (US Patent Application #20120149646) has been filed by Brown University, with JM listed as co-inventor.
