# Supplementary figures and images for: Impairment of TrkB-PSD-95 Signaling in Angelman Syndrome
Source: PLoS Biol. 2013 Feb 12;11(2):e1001478. doi: 10.1371/journal.pbio.1001478 (PMC3570550; doi:10.1371/journal.pbio.1001478)

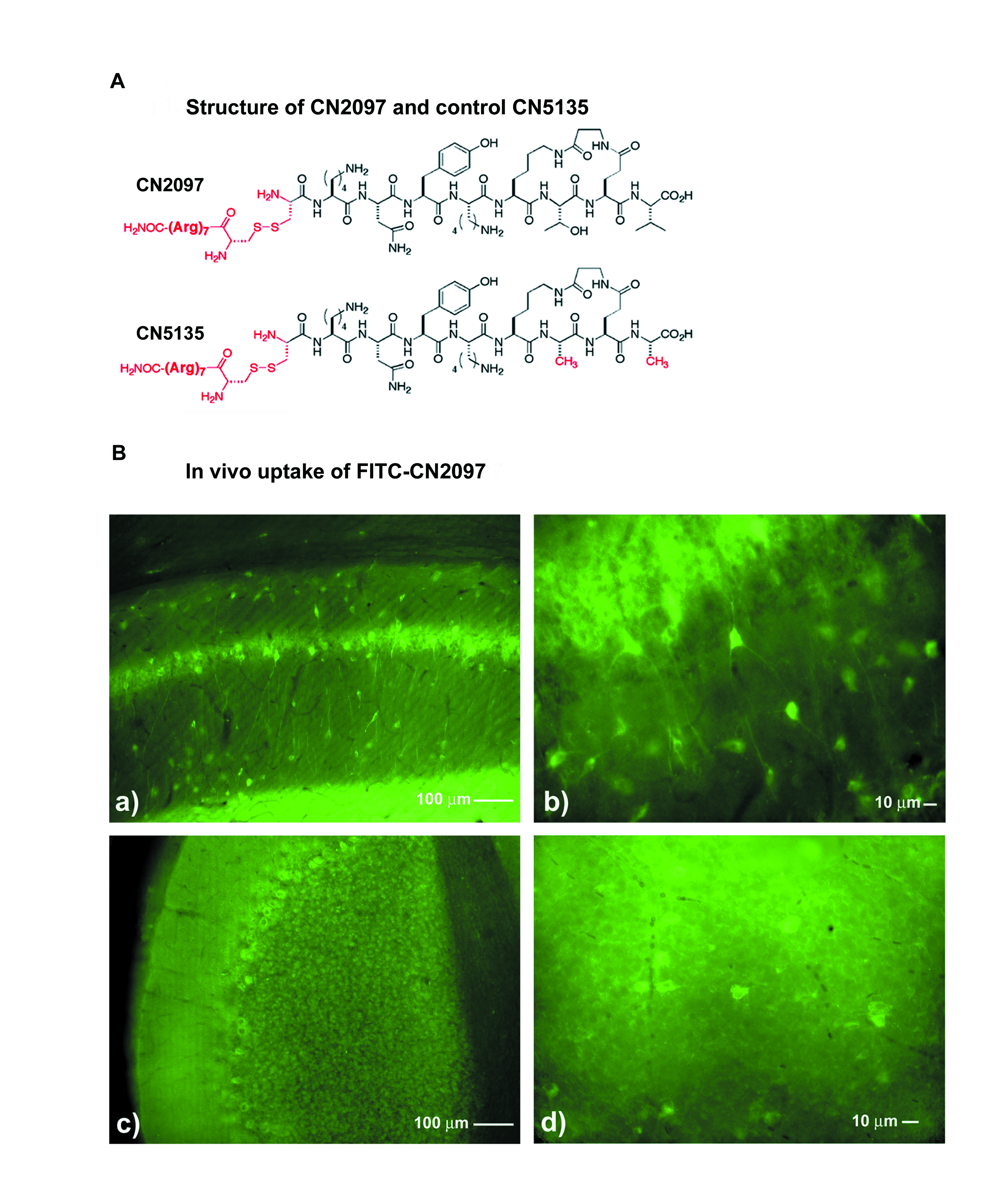

Supplement: Figure S1 — (A) CN2097: Design of a cell-permeable PDZ domain-targeting macrocycle. Standard Fmoc-based protocols [93], were used to synthesize the cyclic-peptide, CN2097, targeting the PDZ domain of PSD-95. The peptide, KNYKKTEV, was cyclized between the Val and Thr residues via a β-alanine linkage and linked to a poly-arginine tail to enhance its uptake by neurons. Also shown is a control cyclic peptide, CN5135, having the Ala/Ala double mutation at the 0/−2 positions, which knocks out binding to PDZ domains. (B) In situ uptake of CN2097 into rat neurons. (A) Pyramidal cells located in CA1 by 24 h following intra-ventricle injection of TMR-tagged CN2097. (B) Similar uptake was noted in multiple populations of rat retinal ganglion cells 6 h following intravitreal injection. (C) Although not as robust as shown in (B), selective uptake in retinal ganglion cells was noted 8 h following an intravenous injection of FITC-tagged CN2097, indicating that the peptide mimic is not only capable of crossing meningeal membrane barriers and selectively taken up by neurons, but also has the ability to cross the blood/brain barrier. (TIFF) [file pbio.1001478.s001.tiff]

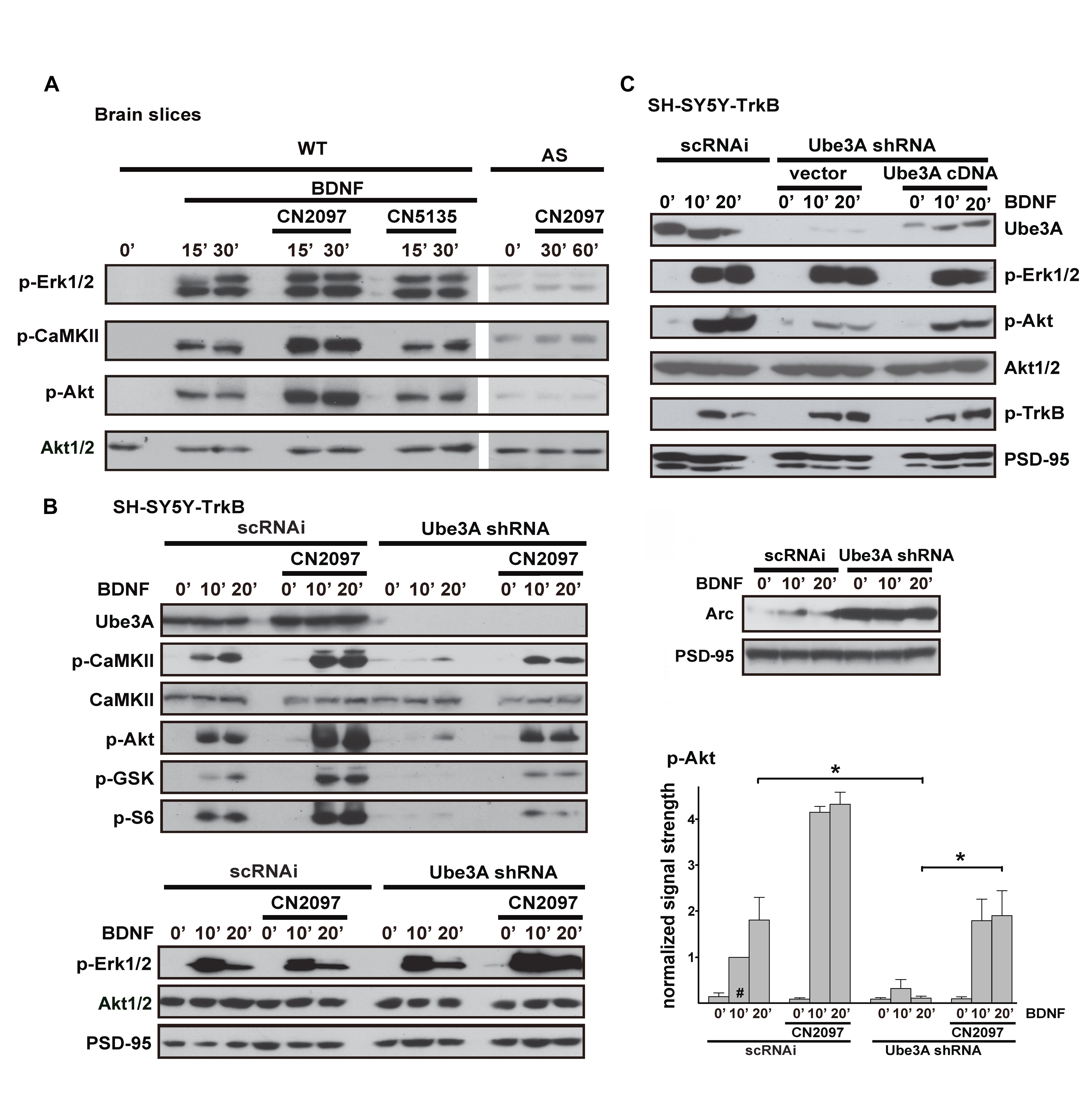

Supplement: Figure S2 — (A) Western blots showing that CN2097 alone does not stimulate Erk, Akt, or CaMKII signaling in AS brain slices, and that the control compound CN5135 does not enhance BDNF signaling over levels produced by BDNF alone in WT slices. WT slices exposed to CN2097, show a 2- to 3-fold enhancement of BDNF-induced p-Akt and p-CaMKII signaling (p<0.01), over slices treated with BDNF alone. The level of basal p-CaMKIIα (phospho-Thr286) is enhanced in AS untreated slices compared to WT (0′, lanes indicated with an #). Blots were probed for p-Erk1/2, p-CaMKII, p-Akt-S473, and Akt loading control. WT slices were stimulated with BDNF (50 ng/ml) in the presence or absence of CN2097 (2 µM), or the control compound CN5135 (2 µM; 30-min pretreatment). AS brain slices were treated with CN2097 (2 µM). (B) CN2097 improves BDNF signaling in Ube3A knockdown SH-SY5Y-TrkB cells. Upper panel: Western blot analysis of protein lysates prepared from TrkB transfected SH-SY5Y cells (SH-SY5Y-TrkB) cotransfected with scrambled control shRNA (scRNAi) or Ube3A shRNA (48 h), treated with BDNF (25 ng/ml) in the presence or absence of CN2097 (2 µM, 20-min pretreatment). Expression of Ube3A, p-CaMKII, p-Akt, p-GSK, p-S6, and CaMKII (sister gel) are shown. Knockdown of Ube3A impeded BDNF-induced p-CaMKII, p-Akt, p-GSK, and p-S6 activation. Lower panel: Depleting Ube3A expression using Ube3A shRNA did not alter BDNF induced pErk activation compared to scrambled RNAi control. Akt1/2 and PSD-95 levels were also not affected by Ube3A knockdown and serve as loading controls. Middle right panel: Knockdown of Ube3A enhances Arc expression in SH-SY5Y cells. SH-SY5Y-TrkB cells transfected with scRNAi or Ube3A shRNA show increased Arc levels in Ube3A-depleted cells, whereas PSD-95 levels were unaffected. Lower right panel: Quantitation of the p-Akt western blot lanes (left panel). Knockdown of Ube3A results in a significant loss of p-Akt (p<0.05) and CN2097 restores p-Akt levels to control levels (p>0.1). # indicat [file pbio.1001478.s002.tiff]

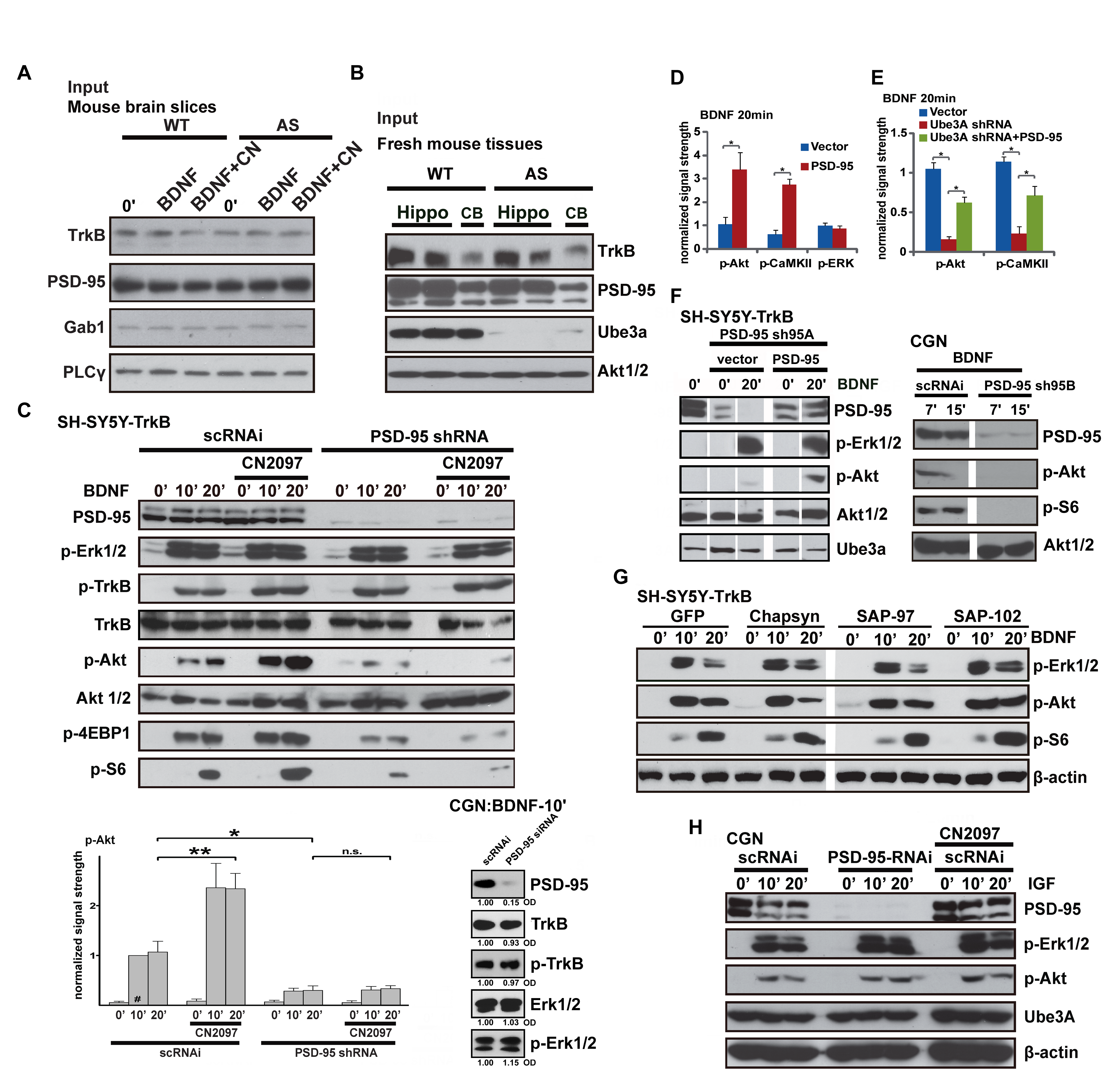

Supplement: Figure S3 — (A) The input lanes of lysate used for WT and AS slices TrkB-coIP westerns shown in Figure 3A (upper panel) show the expression level of TrkB, PLCγ, PSD-95, and Gab1. CN, CN2097. (B) The input lanes of lysate from fresh hippocampus (hippo) and cerebellum (CB) of WT and AS, used for TrkB co-IP Western blots shown in Figure 3B, show the expression level of TrkB, PSD-95, Ube3A, and Akt1/2. (C) Knockdown of PSD-95 in SH-SY5Y-TrkB cells disrupt CN2097 enhancement of BDNF signaling. Upper panel: Western blot analysis of protein lysates prepared from TrkB transfected SH-SY5Y cells (SH-SY5Y-TrkB, 48 h) cotransfected with scrambled control shRNA (scRNAi) or PSD-95 shRNA (sh95A), treated with BDNF (25 ng/ml) in the presence or absence of CN2097 (2 µM, 20-min pretreatment). The expression level of PSD-95 and phosphorylation of Erk, TrkB, Akt, 4E-BP1, and S6 were detected. TrkB was generated from an identically loaded sister gel. The RNAi mediated knockdown of PSD-95 reduced expression to 14.1%±3.5% compared to untransfected cells, and impeded BDNF-induced p-Akt, p-4E-BP1, and p-S6 induction as compared with cells transfected with scRNAi. Furthermore, CN2097 could not rescue BDNF signaling in the PSD-95 depleted cells. Lower left panel: Quantitation of Western blot data for p-Akt. CN2097 significantly enhances p-Akt signaling (**p<0.01). Knockdown of PSD-95 blocks p-Akt signaling and prevents CN2097 rescue of signaling (p>0.1). # indicates column that is normalized to 1.0; ns, not significant. Lower right panel: PSD-95 RNAi knockdown in CGNs does not change the levels of TrkB expression or inhibit BDNF-induced TrkB activation. Western blot analysis of PSD-95, TrkB and ERK expression, and phosphorylation of Erk and TrkB. Primary mouse CGNs (p5) transfected with scrambled control (scRNAi, 200 nM, 24 h) or PSD-95 siRNA oligonucleotides (sh95A; 200 nM, 24 h) were serum starved and treated with BDNF (25 ng/ml, 10 min). Numbers below selected rows represent normalized optical density [file pbio.1001478.s003.tiff]

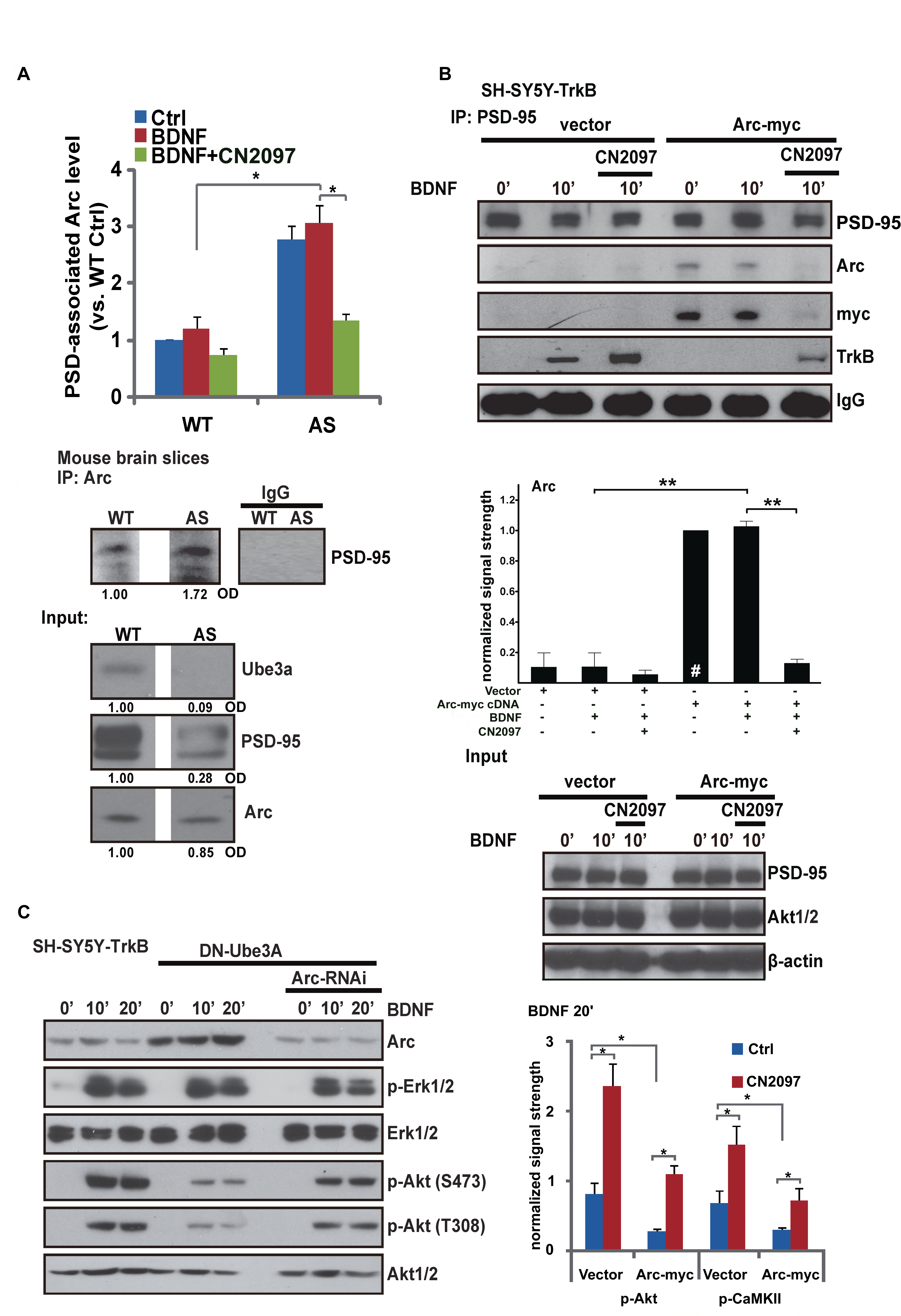

Supplement: Figure S4 — (A) Upper panel: quantitation for the association of Arc and PSD-95 in Figure 4A. AS mice have significantly greater levels of Arc associated with PSD-95 than their WT counterparts (*p<0.05) and CN2097 disrupts this (*p<0.05). Middle panel: Arc and PSD-95 show greater association in AS mice after normalization of the input of Arc. Co-IP assay with an antibody to Arc from lysates prepared from both WT (1 mg lysate) and AS (0.33 mg lysate; note AS Arc input is 0.85 of WT). Western blots were probed with antibodies to PSD-95 show that the association of PSD-95 with Arc is significantly greater in the AS mouse (p<0.05). Lower panel shows the input levels of Arc, Ube3A and PSD-95 detected in lysates normalized for Arc. Numbers below selected rows represent normalized optical density (OD) values. (B) Arc associates with PSD-95 to disrupt TrkB-PSD-95 association. Upper panel: Co-IP assay with an antibody to PSD-95 from lysates prepared from TrkB transfected SH-SY5Y cells (SH-SY5Y-TrkB, 48 h) cotransfected with control empty vector or Arc-myc-tagged cDNA. Cells were untreated or stimulated with BDNF (25 ng/ml, 10 min) in the presence or absence of CN2097 (2 µM, 20-min pretreatment). Western blots were probed with antibodies to Arc, Myc, PSD-95, and TrkB. Bar graph: Quantitation of the association of Arc and PSD-95 (n = 3) represented in the upper panel. Arc transfection resulted in a significant increase in its association with PSD-95 (**p<0.01), and disrupted TrkB binding. CN2097 prevented Arc association with PSD-95 (**p<0.01), and restored TrkB binding to WT levels (p<0.1). Middle lower right panel: The input lanes for each treatment showing equal expression and loading of PSD-95, Akt, and β-actin. Lower right panel: Quantitation of Figure 4B showing that Arc expression results in significantly lower BDNF-induced p-Akt and p-CaMKII (p<0.05). BDNF-signaling in SH-SY5Y-TrkB cells co-transfected with either null-vector or Arc-myc containing vector (acquired from Western blo [file pbio.1001478.s004.tiff]

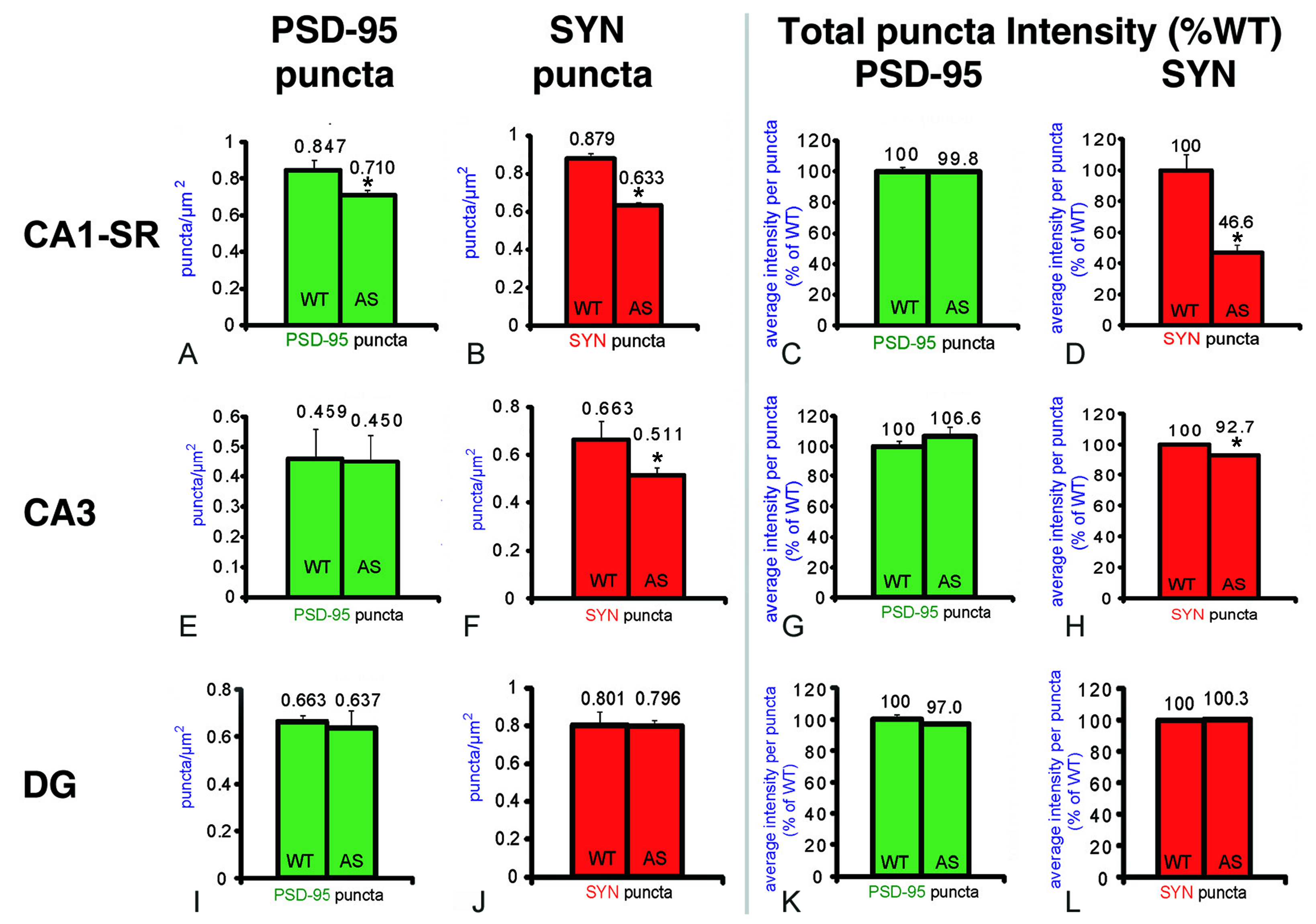

Supplement: Figure S5 — PSD-95 (green) and SYN (red) antibodies were used to stain synapses in the hippocampus. PSD-95 and SYN stained puncta quantified using image J are displayed for CA1-SR (A–D), CA3 (E–H), and DG (I–L) and showing relative distributions of stained puncta and the average staining intensities of the identified puncta between WT and AS mice. Results show that in CA1-SR there is a significant decline in the relative number of PSD-95 stained puncta in the AS mouse (A), whereas no differences were recorded in CA3 (E) and in the DG (I). Similarly, there was a significant decline in the number of SYN-puncta noted in CA1 (B) and also in CA3 (F) but not in the DG (J). In comparing the average relative staining intensities of the captured puncta, there were no differences noted between WT and AS for PSD-95 for all three regions sampled (C, G, and K); however, significant differences in the average staining intensity for SYN were recorded in CA1 (D) and CA3 (H), but not in the DG (L). Immunostained sections were imaged on a confocal microscope using 100× objective and a 4× zoom. Scale bar = 7.5 µm. (TIFF) [file pbio.1001478.s005.tiff]
